# Do Human Reinforcement Learning Models Account for Key Experimental Choice Patterns in the Iowa Gambling Task?

**DOI:** 10.1007/s42113-024-00228-2

**Published:** 2024-11-07

**Authors:** Sherwin Nedaei Janbesaraei, Amir Hosein Hadian Rasanan, Vahid Nejati, Jamal Amani Rad

**Affiliations:** 1 Institute for Cognitive Sciences Studies (ICSS), Tehran, Iran; 2 Department of Psychology, University of Basel, Missionsstrasse 62A, 4055 Basel, Switzerland; 3 Department of Psychology, Shahid Beheshti University, Tehran, Iran; 4 Choice Modelling Centre and Institute for Transport Studies, University of Leeds, LS2 9JT Leeds, UK

**Keywords:** Choice patterns, Cognitive modeling, Iowa gambling task, Model comparison, Parameter space partitioning, Reinforcement learning, Risky decisions

## Abstract

The Iowa gambling task (IGT) is widely used to study risky decision-making and learning from rewards and punishments. Although numerous cognitive models have been developed using reinforcement learning frameworks to investigate the processes underlying the IGT, no single model has consistently been identified as superior, largely due to the overlooked importance of model flexibility in capturing choice patterns. This study examines whether human reinforcement learning models adequately capture key experimental choice patterns observed in IGT data. Using simulation and parameter space partitioning (PSP) methods, we explored the parameter space of two recently introduced models—Outcome-Representation Learning and Value plus Sequential Exploration—alongside four traditional models. PSP, a global analysis method, investigates what patterns are relevant to the parameters’ spaces of a model, thereby providing insights into model flexibility. The PSP study revealed varying potentials among candidate models to generate relevant choice patterns in IGT, suggesting that model selection may be dependent on the specific choice patterns present in a given dataset. We investigated central choice patterns and fitted all models by analyzing a comprehensive data pool (*N* = 1428) comprising 45 behavioral datasets from both healthy and clinical populations. Applying Akaike and Bayesian information criteria, we found that the Value plus Sequential Exploration model outperformed others due to its balanced potential to generate all experimentally observed choice patterns. These findings suggested that the search for a suitable IGT model may have reached its conclusion, emphasizing the importance of aligning a model’s parameter space with experimentally observed choice patterns for achieving high accuracy in cognitive modeling.

## Introduction

Decision-making is a complex cognitive process that integrates learning, emotions, and choice behavior. Understanding how humans make decisions, particularly under uncertainty, has been a central focus in cognitive science. One of the most influential experimental paradigms for studying decision-making under uncertainty is the Iowa gambling task (Bechara et al., 1994; Brevers et al., 2013), which simulates real-life decision-making by requiring participants to choose from decks of cards with varying levels of reward and punishment. Over time, optimal performance requires recognizing and avoiding decks that yield larger penalties in favor of those with smaller but more consistent rewards (Beitz et al., 2014; Erev and Barron, 2005; Worthy et al., 2013). This task has been widely used to explore the cognitive and emotional mechanisms underlying decision-making in both healthy individuals (Beitz et al., 2014; Steingroever et al., 2013, 2015; Overman et al., 2006; van den Bos et al., 2013; Singh, 2016) and clinical populations (Schuermann et al., 2011; Busemeyer et al., 2003; Chan et al., 2014; Palminteri et al., 2012; Cavedini et al., 2002a; Premkumar et al., 2008; Poletti et al., 2011; Stout et al., 2004; Brevers et al., 2014), particularly in patients with impaired executive function (Bechara et al., 1994, 1997, 2000; Brevers et al., 2013).

In recent decades, the intersection of psychology and computational modeling has given rise to the field of computational psychology (Lewandowsky and Farrell, 2011; Wilson and Ten Collins, 2019; Farrell and Lewandowsky, 2010; Johnson, 2006; Busemeyer and Stout, 2002). This discipline uses mathematical models to rigorously analyze and formalize cognitive processes, offering a quantitative approach to hypothesis testing and theory comparison. Computational models, particularly those based on reinforcement learning (RL) (Frank and Claus, 2006; Fontanesi et al., 2019; Kalidindi and Bowman, 2007; Agay et al., 2010; Eckstein et al., 2021; Schwenck, 2017), have proven invaluable in deconstructing the cognitive mechanisms involved in tasks such as the IGT. These models allow researchers to link observed behaviors, such as choices and response times, to underlying cognitive processes such as reward sensitivity, learning rates, and risk preferences, as they simulate how individuals adapt their choices based on past outcomes (Busemeyer and Stout, 2002; Ahn et al., 2008, 2013; Steingroever et al., 2013a; Haines et al., 2018; Ligneul, 2019; Hadian Rasanan et al., 2024; Ghaderi et al., 2024a, b).

Over the years, many models have been developed to explain IGT performance, each aiming to account for different aspects of human decision-making (Busemeyer and Stout, 2002; Ahn et al., 2008, 2013; Steingroever et al., 2013a; Haines et al., 2018; Ligneul, 2019). However, comparisons across models often lead to mixed results, with no clear consensus on which model best captures the complexity of IGT behavior (Steingroever et al., 2013b, a; Haines et al., 2018; Ligneul, 2019). Furthermore, many studies focus solely on fitting models to data without exploring the range of behaviors each model can generate (Busemeyer and Stout, 2002; Yechiam and Busemeyer, 2005; Ahn et al., 2008; Fridberg et al., 2010; Haines et al., 2018; Ligneul, 2019; Yechiam et al., 2008). This limitation calls for a more comprehensive evaluation of models that considers both model fit and the breadth of behavioral patterns they can explain.

In this paper, we aim to address this gap by comparing several reinforcement learning models, including more recently proposed models, to determine which best explains behavior in the IGT. Specifically, we compare two key models: the Outcome-Representation Learning (ORL) model, developed by Haines et al. (2018), and the Value plus Sequential Exploration (VSE) model, introduced by Ligneul (Ligneul, 2019). These models provide unique insights into the decision-making process by focusing on different mechanisms that shape behavior in the IGT.

The ORL model captures four key mechanisms of decision-making. First, it separates the evaluation of gains and losses, allowing for distinct sensitivity to positive and negative outcomes. Second, it explicitly accounts for the frequency of wins, reflecting the preference for decks with frequent wins despite lower overall expected value-a nuance often missed by simpler models (Ahn et al., 2008). Third, it models choice perseverance, representing the trade-off between sticking with a current choice and exploring new ones. Lastly, the ORL model introduces a mechanism for reversal learning, where individuals switch preferences after encountering significant losses, a common behavior observed in IGT participants (Steingroever et al., 2014).

On the other hand, the VSE model delves into how individuals navigate the exploration-exploitation dilemma by combining gain and loss magnitudes into a nonlinear utility function. This model distinguishes between two types of exploration: directed exploration, where individuals sequentially sample different decks, and random exploration, where choices appear more spontaneous (Ligneul, 2019; Wilson et al., 2014). The VSE model also incorporates an exploration bonus parameter, which adjusts the tendency to explore based on past outcomes. Unlike the ORL model, the VSE model emphasizes sequential exploration and the dynamic balancing of both exploration and exploitation strategies.

Our primary contribution lies in the comprehensive comparison of these models using data from 45 studies. By evaluating how well these models capture various aspects of decision-making in the IGT, we aim to shed light on their relative strengths and weaknesses, offering a clearer understanding of which cognitive processes they best explain. This comparison is crucial, not only for understanding decision-making mechanisms but also for improving the reproducibility and generalizability of findings across different studies and populations.

Moreover, the other key contribution of this work is the use of parameter space partitioning (PSP; Pitt et al., 2006;Pitt et al., 2008;Steegen et al., 2017) as a tool to evaluate models. PSP allows us to examine the range of behaviors that each model can potentially generate across its parameter space, offering insights beyond traditional goodness-of-fit metrics such as the Bayesian information criterion (BIC) (Neath and Cavanaugh, 2012) and Akaike information criterion (AIC) (Bozdogan, 1987). This method provides a more nuanced understanding of model performance by highlighting the regions of parameter space where each model excels or fails. This approach reveals whether a model’s success in fitting observed behavior is due to a broad capacity to capture various strategies or limited to a narrow parameter range. Our findings suggest that considering both model fit and the diversity of behaviors a model can simulate is crucial for evaluating the utility of computational models. Additionally, the study considers multiple criteria for model evaluation, including goodness-of-fit measures such as AIC, BIC, and Watanabe-Akaike information criterion (WAIC) (Gelman et al., 2014), to offer a comprehensive assessment of model validity.

**Table 1:** Summary of the payoff scheme of the original version of IGT, developed by Bechara et al. (1994)

	A	B	C	D
Reward per trial	100	100	50	50
Loss per 10 cards	$-$1250	$-$1250	$-$250	$-$250
Number of losses per 10 cards	5	1	5	1
Net outcome per 10 cards	$-$250	$-$250	250	250

Decks A and B are considered disadvantageous because both yield a net outcome equal to $-250$ per 10 cards. While Deck A has frequent losses, $5 \times -250$ losses per 10 cards, Deck B has infrequent losses, with one loss equal to $-1250$. Decks C and D are considered advantageous, where the net outcome per 10 cards for both these Decks equals 250. While Deck C has frequent losses ( $5 \times$ $-50$), Deck D has only one loss equal to $-250$

We also provide a detailed analysis of the role of different model parameters, particularly in the ORL and VSE models, and how they relate to observable patterns in IGT data. This analysis highlights which behavioral strategies are favored by each model and how they align with empirical observations from previous IGT studies. Additionally, we explore the implications of different parameter ranges, discussing how these choices influence the models’ ability to generalize to unseen data.

By integrating these approaches, the present study aims to contribute to the ongoing debate about the best computational models for the IGT and, more broadly, to enhance our understanding of the cognitive mechanisms underlying decision-making processes. The insights gained from this work could have significant implications for clinical psychology, particularly in understanding and treating decision-making deficits in various populations and the factors that drive individuals to make risky or cautious decisions in uncertain environments.

The remainder of this paper is organized as follows: We first review the most relevant models in the literature, focusing on RL-based models that have been applied to the IGT. Next, we describe the ORL and VSE models in detail, followed by a discussion of the parameter space partitioning methodology and the criteria used to compare models. Finally, we present the results of our model comparison and discuss their implications for future research on decision-making models in the IGT.

## Background and Literature Review

### Iowa Gambling Task

The IGT (Bechara et al., 1994) involves four decks of cards (A, B, C, D), and participants are asked to choose cards individually. The task ends after the 100th card is chosen, but participants are unaware of how many trials are left. Decks A and B offer a reward of +100 for each card chosen, but they also come with potential losses totaling $-$1250 for every 10 cards. Deck A has frequent but smaller losses, while Deck B has infrequent larger losses. Both decks result in a net loss of $-$250 for every 10 cards. Decks C and D, however, provide a smaller reward of +50 for each card chosen. Deck D has infrequent significant losses of $-$250 for every 10 cards, while Deck C has frequent smaller losses of $-$50. Decks C and D have a net profit of +250 for every 10 cards. Decks A and B are considered disadvantageous or “bad” due to the negative long-term expected value. In contrast, Decks C and D are considered advantageous or “good” due to the positive overall outcome. The goal of the task is to maximize the final net profit, and participants can switch between decks based on their preferences. Several modified versions of the IGT have been proposed by different researchers, each with its variations and enhancements (Proctor et al., 2014; Stocco et al., 2009; Chiu and Lin, 2007; Lawrence et al., 2009). A summary of the IGT pay scheme for every 10 trials is provided in Table 1.

The task’s simplicity and its ability to mimic real-world decision-making have led to its widespread adoption across various clinical populations. For instance, Cavedini et al. (2002a, 2002b) used IGT for patients with obsessive-compulsive disorder, panic disorder, and pathological gambling clinical groups. Similarly, multiple researchers have applied IGT to schizophrenia patients (Shurman et al., 2005; Martino et al., 2007; Premkumar et al., 2008; Brambilla et al., 2013), bipolar disorders (Brambilla et al., 2013), and those with Parkinson’s disease (Kobayakawa et al., 2008; Poletti et al., 2011; Evens et al., 2016). Additionally, groups of attention-deficit-hyperactivity disorder in both adults (Toplak et al., 2005; Agay et al., 2010) and children (Garon et al., 2006), as well as psychopathy disorder (Blair et al., 2001), have been other target groups that have been comprehensively evaluated for risky decision-making and learning from experiences with rewards/punishments using IGT. Also, many studies have addressed IGT in substance use disorders, including cocaine (Stout et al., 2004; Tucker et al., 2004; Dom et al., 2005; Verdejo-Garcia et al., 2007; Kjome et al., 2010), heroin (Petry et al., 1998), cannabis (Fridberg et al., 2010; Gonzalez et al., 2012; Vaidya et al., 2012; Moreno et al., 2012), alcohol (Tomassini et al., 2012; Brevers et al., 2014; Kovács et al., 2017; Mazas et al., 2000), and cigarette dependents (Balevich et al., 2013; Durazzo et al., 2018). Moreover, traffic offenders (Lev et al., 2008; Farah et al., 2008), criminals, and inmates were among the groups assessed for decision-making ability by multiple studies (Yechiam et al., 2008; Flórez et al., 2017). Furthermore, extensive and comprehensive studies have also been conducted to assess healthy groups’ performance with IGT, including the works of Steingroever et al. (2013, 2015) and others (Overman et al., 2006; van den Bos et al., 2013; Singh, 2016).

This task involves dealing with uncertainty and considering the consequences of choices, aiming to maximize long-term rewards despite potentially greater short-term gains from disadvantageous options (Brevers et al., 2013). The behavior seen in the IGT reflects real-life decision-making, making it a useful tool for assessing impairments (Schuermann et al., 2011). However, understanding the cognitive mechanisms behind it is challenging due to the complexity of decision-making. The IGT captures the intricate interplay between cognitive and motivational processes (Busemeyer et al., 2003). Cognitive models, based on (neuro)psychological theories, help identify and evaluate these mechanisms when applied to tasks like the IGT, revealing the link between parameters and psychological processes (Busemeyer and Stout, 2002; Beitz et al., 2014; Guest and Martin, 2021; Steingroever et al., 2014; Ahn et al., 2016; Chan et al., 2014). Furthermore, reinforcement learning models are effective for understanding behavior in such complex environments (Erev and Barron, 2005; Worthy et al., 2013).

### Previous Models

Over the past few decades, various computational models have been developed to explain the behavior observed in the IGT, particularly focusing on the underlying cognitive processes such as reinforcement learning. These models have been instrumental in deconstructing the psychological mechanisms involved in decision-making during the task. One of the earliest and most influential is the Expectancy Valence (EV) model, which suggests that individuals form expectancies about the value of each deck and update these expectancies based on their experiences (Busemeyer and Stout, 2002). This model also introduced the idea of a “valence” parameter, which captures (subjects’ attention) sensitivity to gains and losses, marking a foundational step in IGT modeling (Busemeyer and Stout, 2002; Yechiam and Busemeyer, 2005; Yechiam et al., 2005; Steingroever et al., 2013a). A key component of this process is expectancy learning, where individuals develop expectations about the valences associated with each choice, governed by the utility function (Busemeyer and Stout, 2002; Yechiam and Busemeyer, 2005; Yechiam et al., 2005; Steingroever et al., 2013a). This utility function reflects the perceived value of future outcomes, allowing for dynamic adjustment based on reinforcement, further shaping decision-making behavior.

Subsequently, the Prospect Valence Learning (PVL) model expanded on the EV model by incorporating elements from prospect theory, including nonlinear probability weighting and loss aversion (Ahn et al., 2008). The utility function of PVL updates the expectations of all Decks (both selected and not selected Decks) on each trial using a decay-RL rule. This model provided a more nuanced understanding of how people perceive and integrate gains and losses over time, shedding light on the psychological processes driving risk-taking behavior in the IGT (Ahn et al., 2008, 2013; Tversky and Kahneman, 1992).

Building on these foundations, researchers have explored various hybrid models that combine elements of EV and PVL. Notable among these is the EV-PU model, which integrates the utility function from the PVL model while retaining other elements from the EV model (Ahn et al., 2008; Steingroever et al., 2013a). The EV-PU model is characterized by four parameters: shape, loss aversion, recency, and consistency, which are similar to those in the PVL model (Ahn et al., 2008; Steingroever et al., 2013a). This hybrid approach has garnered significant attention for its potential to capture a broader range of decision-making behaviors in the IGT.

Another important hybrid model is the PVL-Delta model, which merges the utility, choice rule, and sensitivity functions from the PVL model with the learning function from the EV model (Fridberg et al., 2010). This model also introduces an updating rate similar to that in the EV model, applying a delta rule to update trial expectations (Fridberg et al., 2010; Ahn et al., 2008; Steingroever et al., 2013b). Like the EV-PU model, the PVL-Delta model is structured around four key parameters: shape, loss aversion, consistency, and updating rate, reflecting a synthesis of the strengths of both EV and PVL models (Fridberg et al., 2010).

A significant advancement was the Value-Plus-Perseveration (VPP) model, which introduced a perseverance component to the valuation process (Worthy et al., 2013). This model addresses a critical flaw in the decay rule of previously proposed RL models-the confusion between the tendency to persist with a choice and the tendency to select the option with the highest expected value (Steingroever et al., 2013a). The VPP model underscored the importance of accounting for both reward-based learning and the tendency to stick with previously made choices, offering a more comprehensive view of the decision-making process (Worthy et al., 2013).

In recent years, more innovative models have been developed to capture deeper cognitive mechanisms. The Outcome-Representation Learning model, proposed by Haines et al. (2018), is particularly noteworthy. The ORL model introduces four key mechanisms: separate evaluation of gains versus losses, explicit consideration of win frequency, choice perseverance, and reversal of learning. These mechanisms offer a more detailed exploration of the cognitive processes involved in the IGT, particularly in how individuals respond to the frequency of wins and losses and how they adjust their strategies following significant losses (Haines et al., 2018). The ORL model thus represents a significant advancement in our understanding of the complex decision-making processes that underlie performance in the IGT.

Among the latest developments is the Value Plus Sequential Exploration model, introduced by Romain Ligneul in 2019 (Ligneul, 2019). This model is distinctive in its use of a nonlinear combination of reward and loss magnitudes during each trial, akin to the utility function in the PVL2 model but without including loss aversion (Ligneul, 2019). The VSE model emphasizes two key strategies to address the exploration-exploitation dilemma: directed exploration, where individuals sequentially sample all decks over consecutive trials, and random exploration. It also introduces an exploration bonus parameter, $\phi$, which reflects a subject’s preference for exploration versus exploitation (Ligneul, 2019). The VSE model, with its sophisticated approach to handling exploration and exploitation, provides a more nuanced framework for understanding decision-making behavior in the IGT.

While these models have advanced our understanding of decision-making in the IGT, they are not without limitations. For instance, comparisons between models have often yielded mixed results, with no clear consensus on which model best captures the complexity of behavior in the IGT (Steingroever et al., 2013a, b; Haines et al., 2018). Also, many models assume homogeneity in decision strategies across individuals, potentially oversimplifying the variability observed in human behavior (Steingroever et al., 2013a; Ahn et al., 2008). Furthermore, some models focus primarily on fitting empirical data without adequately exploring the range of behaviors they can simulate, limiting their generalizability and applicability across different populations and settings (Yechiam and Busemeyer, 2005; Fridberg et al., 2010; Haines et al., 2018; Ligneul, 2019).

### Gap Identification

Despite the extensive body of literature on computational models for the Iowa gambling task, several gaps remain. One significant gap is the need for a comprehensive analysis of model flexibility across parameter spaces. Traditional model comparisons often rely on metrics such as the AIC, BIC, or WAIC, which primarily focus on goodness-of-fit. However, these measures do not fully capture the range of behaviors that a model can generate, limiting our understanding of a model’s flexibility and generalizability across different contexts.

Another gap lies in the cross-validation of these models across diverse populations and settings. While many studies have focused on specific groups, such as healthy controls or clinical populations, there has been insufficient exploration of how well these models generalize to other contexts. This limitation underscores the importance of conducting a global analysis that not only evaluates model fit but also examines the robustness and flexibility of models across different experimental conditions.

Furthermore, recent developments in computational modeling, particularly those based on reinforcement learning, have not been fully integrated into the analysis of IGT performance. These newer models, such as the ORL and VSE models, offer a more dynamic perspective on decision-making by capturing how individuals adapt their strategies over time based on their experiences. However, these models have not been widely compared with earlier models, and their full potential in explaining IGT performance has yet to be realized.

In earlier comparisons of cognitive models, researchers employed several methods, such as post hoc fit criteria (Busemeyer and Stout, 2002; Yechiam and Busemeyer, 2005; Ahn et al., 2008; Fridberg et al., 2010; Haines et al., 2018; Ligneul, 2019) to predict choices based on previous trials, generalization criteria (Ahn et al., 2008; Yechiam and Busemeyer, 2005; Yechiam et al., 2008; Haines et al., 2018) to assess model predictions using parameters estimated from different tasks, and simulation techniques (Ahn et al., 2008; Fridberg et al., 2010; Steingroever et al., 2014; Haines et al., 2018; Ligneul, 2019) that generated choices using best-fitting parameters rather than real choice information. However, these studies have yielded inconsistent results, and no single model consistently emerged as superior in all scenarios (for a detailed understanding of these discrepancies in the drawn conclusions, see Steingroever et al. (2013a, 2013b)). For instance, Haines et al. found that the ORL model, despite having fewer parameters, performed comparably to the VPP model in short-term prediction and outperformed the PVL-Delta model in terms of parameter recovery (Haines et al., 2018). However, they could not determine which model was superior for long-term prediction using choice simulation. In contrast, Romain Ligneul’s study showed that the VSE model outperformed ORL, EV, PVL, PVL-Delta, and VPP models using fitting accuracy metrics such as BIC and AIC. Additionally, the VSE model demonstrated superior parameter recovery (Ligneul, 2019).

While the VSE model has often demonstrated superior performance in fitting participant data across different groups, it does not always outperform other models, particularly when results from various datasets are taken into account. To understand why VSE falls short in these cases, it is crucial to assess the additional capabilities and limitations of other models.

A significant factor in these inconsistencies is the diversity of choice strategy patterns in the data. Participants in the IGT exhibit different choice strategies, and each model is designed to capture a subset of these, in the model’s assumptions. In general, these strategies result in the following choice patterns (Steingroever et al., 2013a):

- **Good-Over-Bad (GOB):** Favoring cards from advantageous Decks (C and D) over disadvantageous ones (A and B).
- **Bad-Over-Good (BOG):** Favoring cards from disadvantageous Decks over advantageous ones.
- **Infrequent-Over-Frequent (IOF):** Preferring Decks with a lower frequency of losses (B and D) over those with a higher frequency of losses (A and C).
- **Frequent-Over-Infrequent (FOI):** Preferring Decks with higher loss frequency over those with lower loss frequency.

Each model is equipped with multiple parameters and thus has the potential to generate different choice patterns. However, some models may generate choice patterns that do not align with experimental data for a significant portion of their parameter space. This reduces the model’s ability to capture the full spectrum of strategies exhibited by participants. A model that consistently produces a specific choice pattern across most of its parameter space may be well-suited for datasets that exhibit that pattern but will struggle with others. Therefore, it is essential for a model’s parameter space to align well with experimentally observed patterns and be balanced for the sake of generalizability (Steingroever et al., 2013a).

To address these gaps, PSP has emerged as a powerful tool for evaluating model flexibility. PSP generates all possible choice patterns for a model and computes the proportions of those patterns within the model’s parameter space, providing a more robust understanding of how well a model captures the range of observed behaviors (Pitt et al., 2006, 2008; Steegen et al., 2017).

Steingroever and colleagues applied the PSP method to assess the EV, PVL, EV-PU, and PVL-Delta models (Steingroever et al., 2013a, b). Their results revealed that these models struggle to produce commonly observed choice patterns. For instance, while the “good-over-bad” pattern is central to all models, the EV model performed poorly when capturing the “infrequent-over-frequent” pattern. Conversely, the PVL, EV-PU, and PVL-Delta models performed poorly on the “bad-over-good” pattern. As a result, Steingroever and colleagues concluded that traditional RL models were insufficient in explaining key psychological processes underlying the IGT, advocating for the development of improved models (Steingroever et al., 2013a, b).

The question remains: Is there an optimal model for the IGT that effectively addresses the limitations of earlier RL models? To answer this, it is necessary to conduct an in-depth evaluation of the flexibility of newer models, such as ORL and VSE, and compare them with older RL models using the PSP method. By doing so, this study aims to determine whether these novel models offer meaningful improvements in capturing diverse choice patterns and generalizing across different datasets, providing clearer guidance for future research.

## Computational Models of the Iowa Gambling Task

As previously discussed, several traditional RL models have been developed to explain decision-making behavior in the Iowa gambling task. These models, such as EV, PVL, and their hybrid extensions, form the foundational basis of computational modeling in this domain. We have already covered the conceptual aspects of these models, including their theoretical mechanisms and relevance to decision-making in the IGT. However, to streamline the main manuscript and maintain a clear focus on our primary contributions, we have moved any remaining technical details-such as model equations, parameter definitions, and extended discussions on model behavior-to Appendix A for interested readers.

In this section, we focus on two recent and advanced models: the ORL and VSE models, which offer novel perspectives on decision-making in the IGT.

**Table 2:** Equations utilized in the ORL model (Haines et al., 2018) and the corresponding parameters

Functions	Equations	Parameters	Range
Expected value	$$EV_{j}(t+1)= \left\{ \begin{array}{ll} EV_{j}(t)+A_{rew}.(x(t)-EV_{j}(t)),~~~\textrm{if}~ x(t)\geqslant 0 \\ EV_{j}(t)+A_{pun}.(x(t)-EV_{j}(t)),~~~otherwise \end{array}\right. $$	$$\begin{array}{ll} A_{rew}:\\ \text {Reward}/\text {positive learning rate}\\ A_{pun}:\\ \text {Punishment}/\text {negative learning rate} \end{array}$$	$$\begin{array}{ll} (0,1) \\ \\ (0,1) \end{array}$$
$$\begin{array}{ll}Win frequency\\ (Chosen Deck) \end{array}$$	$$EF_{j}(t+1)=\left\{ \begin{array}{ll}EF_{j}(t)+A_{rew}.(sgn(x(t))-EF_{j}(t)),~~~\textrm{if}~x(t)\geqslant 0\\ EF_{j}(t)+A_{pun}.(sgn(x(t))-EF_{j}(t)),~~~otherwise \end{array}\right. $$		
$$\begin{array}{ll}Win frequency\\ (Unchosen Deck)\end{array}$$	$$EF_{j'}(t+1)=\left\{ \begin{array}{ll}EF_{j'}(t)+A_{rew}.(\frac{-sgn(x(t))}{c}-EF_{j'}(t)),~~~\textrm{if}~x(t)\geqslant 0\\ EF_{j'}(t)+A_{pun}.(\frac{-sgn(x(t))}{c}-EF_{j'}(t)),~~~otherwise\end{array}\right. $$		
Choice perseverance	$$PS_{j}(t+1)=\left\{ \begin{array}{ll}\frac{1}{1+K},~~~if~D(t)=j\\ \frac{PS_{j}(t)}{1+K},~~~otherwise\end{array}\right. $$ , where $K=3^{K'}-1$	K: Decay parameter	$$\begin{array}{ll}K'\in [0,5] \\ K\in [0,242]\end{array}$$
Single value signal	$V_{j}(t+1)=EV_{j}(t+1)+EF_{j}(t+1).\beta _{F}+PS_{j}(t+1).\beta _{P}$	$$\begin{array}{ll}\beta _{F}:\\ \text {Outcome frequency weight}\\ \beta _{P}: \\ \text {Perseverance weight} \end{array}$$	$(-\infty ,\infty )$

Note that $X(t) = W(t) - L(t)$, is the net outcome, where *W*(*t*) represents the money won and *L*(*t*) represents the money lost at time *t*. Also, *c* is the number of choices other than the chosen deck. In the original version of the IGT, where there are four decks to choose from, *c* is fixed at 3

### Outcome-Representation Learning Model

Recent innovations have introduced computational reinforcement learning models for the Iowa gambling task that explore deeper cognitive mechanisms. The *Outcome-Representation Learning* model, proposed by Haines et al. (2018), stands out with its four basic design mechanisms:

- **Separate Evaluation of Gains vs. Losses:** The first mechanism of ORL captures a separate evaluation of gains versus losses, with potential implications in real-world scenarios (Haines et al., 2018).
- **Frequency of Wins:** This mechanism explicitly considers the frequency of wins, addressing the preference for Decks with higher win frequencies despite the long-term expected value, as noted by Lin et al. (2007), a nuance not captured by the delta learning rules for expected value and loss aversion in the PVL model.
- **Choice Perseverance:** The third mechanism that ORL aims to capture is the preserved tendency of subjects to either stick with one Deck or switch between Decks (exploration and exploitation dilemma), which seems to be stable over time among healthy participants (Steingroever et al., 2013).
- **Reversal of Learning:** The fourth mechanism is the reversal of learning, where the preference for a Deck reverses after a significant loss—precisely, the reversal of preference for Deck B—which many participants initially prefer due to its high win and low loss frequencies (Steingroever et al., 2014).

Table 2 summarizes the mathematical underpinnings of these mechanisms. In this model, the expected value for a selected Deck denoted as *j* is directly updated based on the objective outcome:

- **Gains:** If the outcome results in a gain, then the difference between the outcome on trial *t* and the expected value of Deck *j* on trial *t* is multiplied by a learning rate $0<A_{rew}<1$, which signifies sensitivity to the received reward.
- **Losses:** If the outcome is a loss, the parameter $0<A_{pun}<1$ controls the sensitivity to that loss.

A more significant difference between these two parameters suggests heightened sensitivity to either gain or loss.

The second mechanism here involves the win frequency of both chosen and unchosen Decks, which are updated using the same delta rule but with minor differences and are controlled by the same parameters $0<A_{rew}<1$ and $0<A_{pun}<1$ (see Table 2) the *sgn*(*x*(*t*)) yields $-$1, 1, and 0 for negative, positive, and zero net outcomes, respectively. It should be noted that a negative value of *sgn*(*x*(*t*)) decreases the win frequency of unchosen Decks, divided by the number of unchosen Decks (three in the IGT context), and is added to the last expected frequency of that Deck.

The third mechanism in this model measures choice perseverance, regardless of the outcome. It is governed by a single parameter: the decay parameter *k*. The decay parameter measures how quickly participants forget past decisions. According to Table 2, the choice perseverance for the chosen deck—the same choice as the last trial—equals one divided by the decay parameter + 1. For other decks, it is updated by dividing the last value by the decay parameter + 1, indicating a decay over time. Note that $K = 0$ implies that participants retain choices in memory for as long as possible, while $K = 242$ suggests they quickly forget the last choice.

Finally, all previous equations are linearly integrated to yield a value signal for each Deck (see the last row in Table 2). The unbounded parameters $\beta _{F}$ and $\beta _{P}$ are two weights that reflect how much the intended value of each Deck is affected by outcome frequency and perseverance. Specifically, $\beta _{F}<0$ indicates a preference for Decks with low win frequency and vice versa, while $\beta _{P}<0$ represents a tendency to switch and $\beta _{P}>0$ indicates a tendency to stick with the previous choice. The only remaining aspect in this model is the selection rule, which is derived using the choice rule of Table 7, where $\theta =1$.

### Value plus Sequential Exploration Model

The most recent model proposed for the Iowa gambling task is the *Value plus Sequential Exploration*, introduced by Romain Ligneul in 2019 (Ligneul, 2019). This model is distinctive because it uses five parameters different from those used in the ORL model. The VSE model assumes individuals utilize a nonlinear combination of reward and loss magnitudes during each trial. This assumption is similar to the utility function previously introduced in the *prospect valence learning 2 (PVL2)* model (Dai et al., 2015), but it does not include loss aversion. The parameter $\theta$ in the VSE model represents sensitivity and functions similarly to the shape parameter (parameter *A* in Table 7) in the PVL, influencing choice sensitivity comparably. This model is built upon two key strategies to address the exploration-exploitation dilemma: directed and random exploration (Wilson et al., 2014).

Directed exploration refers to a tendency where all Decks are sampled sequentially over four consecutive trials, a choice pattern that seems to occur more frequently than by mere chance (Ligneul, 2019).

#### Updating Weights

The model suggests that when the subject combines rewards and losses, they update the exploration and exploitation weights separately for chosen and unchosen Decks. For the chosen Deck, the exploitation weight for the subsequent trial is calculated as the sum of the net outcome (reward minus loss, factoring in sensitivity) and the decayed value of the previous exploitation value of that Deck. However, the exploitation weights of the unchosen Decks do not include the net outcome term. When a Deck’s choice outcome is positive, its exploitation weight increases in proportion to the magnitude of the net outcome while it decays for the other Decks. The exploration weight for the chosen Deck is set to zero, but a delta rule is applied for the unchosen Decks.

#### Exploration Bonus

The exploration bonus ($\phi$) reflects the subject’s preference for exploration. A positive $\phi$ indicates a preference for exploration, while a negative $\phi$ suggests a tendency to stick with the previous Deck (i.e., exploit). This parameter serves as a measure of sequential exploration.

#### Choice Probabilities and Consistency Parameter

A simple softmax equation is employed in all prior models to compute the choice probabilities for all Decks. The consistency parameter *c* (calculated using $c=3^{\beta }-1$, where $0<\beta <5$) determines the extent to which choices are information-driven (for $c>0$) or random (for $c=0$). It is worth noting that $\beta =0$ results in $c=0$, leading to random exploration, while $\beta>>0,~\theta >0$, and $\phi =0$ yield purely value-based exploitation, and $\beta>>0,~\theta =0$, and $\phi >0$ lead to pure directed exploration. A combination of these settings is also possible (Ligneul, 2019). All equations of the VSE model are summarized in Table 3.

**Table 3:** Equations used in the VSE (Ligneul, 2019) model and its related architecture. Note that *d* denotes the deck

Functions	Equations	Parameters	Range
Utility	$v(t)=Gain^\theta (t)-Loss^\theta (t)$	$\theta :$ Sensitivity	[0, 1]
Exploitation	$$Exploit^d(t+1)=\left\{ \begin{array}{ll} Exploit^{d}(t).\Delta +v(t)\quad \text {Chosen Deck} \\ Exploit^{d}(t).\Delta ,\quad \text {Unchosen Decks} \end{array}\right. $$	$\Delta :$ Decay	[0, 1]
Exploration	$$Explore^{d}(t+1)=\left\{ \begin{array}{ll} 0~~~~~~~~~~~~~~, \text {Chosen Deck}\\ Explore^{d}(t)+\alpha .(\phi -Explore^d(t)),~\text {Unchosen Decks}\end{array}\right. $$	$$\begin{array}{ll}\alpha : \text {Learning rate} \\ \phi : \text {Exploration} \\ \text {bonus} \end{array}$$	$$\begin{array}{ll}[0,1]\\ (-\infty ,\infty )\end{array}$$
Choice rule	$P(Choice=d)=\frac{e^{(Explore^{d}+Exploit^{d}).c}}{\sum _{i=1}^{4}e^{(Explore^{i}+Exploit^{i}).c}}~,~~~where~~c=3^{\beta }-1$	$$\begin{array}{ll}c: \text {Consistency} \\ \beta : \text {Inverse} \\ \text {temperature} \end{array}$$	$$\begin{array}{ll}{[}0,242{]}\\ {[}0,5{]} \end{array}$$

## Methods

### Simulation Perspective: PSP

The parameter space partitioning method is a global analysis technique that offers a systematic way to evaluate model flexibility and generalizability (Steegen et al., 2017; Pitt et al., 2008). It was first introduced by Pitt et al. (2006, 2008) to understand the range of behaviors a model can generate, particularly in cognitive modeling. Unlike traditional local analysis methods, such as the post hoc fit criterion or generalization tests that assess a model’s performance at specific parameter values, PSP analyzes the entire parameter space, allowing researchers to understand the full range of predictions a model can make. This broader approach enables researchers to assess not only how well a model fits observed data but also its capacity to generate other plausible patterns that may emerge in different contexts.

A key goal of model evaluation is to determine whether a model can predict different types of observed behaviors. PSP achieves this by dividing the model’s parameter space into distinct regions, where each region corresponds to a specific data pattern. This ability to capture diverse patterns across various datasets is particularly relevant when considering cognitive models for decision-making like the Iowa gambling task, where choice patterns can vary significantly between healthy and clinical populations.

By evaluating the parameter space as a whole, PSP provides insights into the flexibility and generalizability of models. Flexibility refers to the model’s ability to adapt to different types of data and capture a wide range of choice patterns. A model with low flexibility may only explain a limited set of behaviors (choice patterns), which could restrict its applicability across diverse datasets, as real individuals tend to follow strategies leading to at least three distinct choice patterns. Conversely, a highly flexible model is capable of generating a broad range of choice patterns, including those not typically observed in real individuals. From a PSP perspective, an ideal model strikes a fair balance between flexibility and generalizability. A model that limits its flexibility to experimentally observed patterns and fairly distributes its parameter space to the patterns under study is considered a better option. Therefore, a model should be generalizable to unseen data too (Pitt et al., 2008). When it comes to model comparison, it is essential that a candidate model be adequately able to capture all experimentally observed choice patterns.

#### Method Implementation

Applying PSP first requires defining the patterns that the model can predict. It is up to the researcher to define those patterns. In this study, we applied two distinct approaches to define and analyze choice patterns in the IGT: the broad definition and the restricted definition. These definitions serve different purposes and offer unique insights into the decision-making strategies of participants.

*Broad Definition*: The broad definition encompasses all experimentally observed choice patterns, including both common and rare behaviors. This approach allows for the capture of the full spectrum of decision-making strategies, without any a priori assumptions about which patterns are most significant. It is particularly useful when the research aims to explore individual differences and identify a wide range of behaviors, including those that may occur less frequently. Under this definition, we considered five possible choice patterns: *Good-Over-Bad*, *Bad-Over-Good*, *Infrequent-Over-Frequent*, *Frequent-Over-Infrequent*, and *Remaining*. The “Remaining” category includes patterns that do not fit into the other categories and are not empirically observed (Steingroever et al., 2013a).

*Restricted Definition*: The restricted definition, based on the criteria proposed by Steingroever et al. (2013a, 2013b), focuses on the most common and pronounced choice patterns, excluding those that may result from random or less stable behavior. This approach applies stricter criteria to the selection of choice patterns, thereby enhancing the statistical robustness of the analysis. For example, the GOB pattern under the restricted definition requires that the total number of selections from the advantageous decks (C and D) exceeds 65 (i.e., $C + D \ge 65$). By applying these criteria, the restricted definition aims to provide a clearer and more interpretable measure of decision-making tendencies. The simulation step yields a detailed representation of the models’ parameter space, as depicted in Fig. 2.

We utilized the broad definition to conduct a comprehensive analysis of the models’ flexibility and ability to generate diverse choice patterns. However, we also incorporated the restricted definition to ensure that our findings are robust and align with the most established patterns in the literature. The dual application of these definitions allows for a thorough comparison of model performance across different analytical perspectives. By considering both definitions, we ensure that our analysis captures a wide range of behaviors while also maintaining a focus on the most statistically significant patterns. This approach allows for a more balanced and comprehensive evaluation of the models under investigation.

In the next step, the whole parameter space of the model is searched to find out what patterns the model can produce. Originally, a Markov chain Monte Carlo-based method is used to sample points from parameter regions. In a repeated manner, the sampled point and the yielded pattern are recorded. Through this process, if new points do not yield new patterns, then exploring the region stops. However, in this study, we have performed a grid search as suggested by Steingroever et al. (2013a, 2013b). The approach we used is as follows:

For this study, we applied PSP to assess the flexibility of both the ORL and VSE models. Each of these models uses five parameters, and we defined the valid ranges for each parameter based on prior literature. In order to explore the parameter space comprehensively, we conducted a grid search over the entire parameter space. Specifically, we sampled 60 values for each parameter at equal intervals, resulting in a total of $60^{5}$ parameter sets per model.

Each parameter set corresponds to a simulation round in which a simulated participant completes 100 trials of the IGT. The outcome of each simulation is the cumulative number of Decks selected by the participant, and we analyzed the resulting patterns according to both broad and restricted definitions of choice patterns.

#### Evaluating Model Success

A model’s success under PSP is determined by its ability to generate observed choice patterns across a significant portion of its parameter space. This avoids the potential pitfall where a model may appear successful based on a narrow, atypical set of parameters, yet fail to capture most real-world behaviors. A balanced parameter space—where various behavioral patterns are represented equally—indicates high model flexibility and generalizability. Conversely, an unbalanced parameter space that favors certain patterns disproportionately suggests bias and limited generalizability, particularly when applied to diverse datasets.

Through this approach, PSP allows us to systematically evaluate whether a model can capture observed behaviors across a wide range of experimental conditions, providing a more comprehensive assessment than traditional fitting criteria alone.

### Fitting Perspective

#### Experimental Datasets and Participants

The diverse data pool ($N = 1428$) that we have designed here to fit RL models is divided into four broad categories:

- The first category is a data subset, initially created by Steingroever et al. (2015a) through a multi-lab collaboration. This category, now a subset of our comprehensive dataset, comprises data from 10 studies that evaluated 504 healthy participants, none of whom had a psychiatric diagnosis based on DSM criteria. Detailed information about this category, including the number of participants (indicated by ${\textbf {N}}$), the study population (here, the healthy group), and demographic data, can be found in entries one to ten of Table 8. For a more comprehensive understanding and additional details of this category, Steingroever et al.’s work Steingroever et al. (2015a) serves as a valuable reference.
- The second category, which we were able to freely find and add to our data pool, pertains to a significant clinical study by Ahn et al. (2014) This study investigated stimulant and opiate addicts in protracted abstinence, focusing on three groups: heroin users, amphetamine users, and healthy participants. Detailed insights about this category can be found in rows 11 to 13 of Table 8. For an in-depth exploration of this study, including the experimental design and other details, the work of Ahn et al. (2014) is a recommended read.
- The third category encompasses two simulated datasets, denoted as 14 and 15 in Table 8, which were developed by the authors of this study. These datasets were generated through the simulation of the IGT using both ORL and VSE models. Each model was used to mimic the behavior of actual participants in the IGT, involving the sequential selection of cards from the four IGT Decks across 100 trials. From a multitude of simulation attempts, forty were selected to form the GOB and BOG simulated datasets. Each dataset contains twenty samples, with half originating from ORL simulations and the other half from VSE simulations. The GOB simulated dataset was specifically generated to exclude any instances of the Bad-Over-Good pattern. As such, it only includes the Good-Over-Bad and Infrequent-Over-Frequent patterns, in line with the restricted definition and 65 criteria. Similarly, the BOG simulated dataset was carefully curated to eliminate any instances of the Good-Over-Bad pattern. Therefore, it only includes the Bad-Over-Good and Infrequent-Over-Frequent patterns, adhering to the restricted definition and the 65 criteria. The rationale behind such a meticulous construction was to establish a robust and clear test case for assessing the performance of the models in scenarios where the similarity between the patterns in the datasets and the models’ parameter space is amplified.
- The fourth category comprises five distinct clinical studies, all of which have been spearheaded by our research team. The majority of these studies are yet to be published, with some currently in the preparation phase, while drafts of others have already been submitted to various journals for review and potential publication:

$$\blacksquare $$
The study led by Yousefi and Rad (2024), one of five distinct clinical studies in the fourth category, sought to understand the influence of discrete emotions on the cognitive mechanisms that drive risky decision-making. This question was of paramount importance as prior studies have demonstrated that affect biases can influence individuals’ overall performance in risky decision-making tasks, including the adoption of selection strategies among other key decision-making factors. However, there was a knowledge gap regarding how effect influences the specific cognitive mechanisms that underlie decision-making and the identification of the underlying neurocognitive processes (such as hypersensitivity to reward and/or loss, inability to learn from past profits and losses, impulsive response style) that account for the observed performance. Therefore, in the study designed by Yousefi and Rad (2024), 78 healthy individuals (39 women; mean age = 22.46, SD = 3.89) participated in the experiment and were randomly assigned to one of three groups (positive, negative, or neutral mood induction), with each group consisting of 26 individuals. Initially, a Self-Assessment Manikin (SAM) questionnaire was used to assess the mood of the subjects. Subsequently, based on the assigned group, each participant was shown a positive, negative, or neutral mood induction video clip. After viewing the video clip, the SAM questionnaire was administered again to the participants. Finally, to scrutinize the impact of the three emotions—positive, negative, and neutral—on individuals’ decision-making performance, the Balloon Analog Risk Task (BART) and IGT were administered in a counterbalanced manner. In alignment with the objectives of our research, we utilized data from all three mood induction groups in the IGT (refer to lines 16 to 18 of Table 8).
$$\blacksquare $$
The study proposed by Nejati et al. (2024), given the difficulties individuals with General Anxiety Disorder (GAD) have with reward processing, aimed to investigate the dynamics of the ventromedial prefrontal cortex (vmPFC) and dorsolateral prefrontal cortex (dlPFC) activities during decision-making in GAD patients and healthy controls, and the respective alterations induced by transcranial alternating current stimulation (tACS) in the theta frequency range. The study was conducted in a randomized, single-blinded, and complete crossover design. Specifically, seventeen healthy adults and twenty adults with GAD received tACS (1.5 mA, 6 Hz) in 5 separate sessions with the following electrode stimulation protocols: two channels with synchronized stimulation over F3 and FP2, the same electrode placement with desynchronized stimulation, stimulation over F3, or Fp2, and sham stimulation. The return electrode was placed over the contralateral shoulder in all conditions. During stimulation, participants performed the IGT in each session to assess decision-making and learning. In the GAD group, all participants were diagnosed with GAD by a clinical psychologist according to the Diagnostic and Statistical Manual of Mental Disorders 5th ed. (American Psychiatric Association, 2013). All participants had normal or corrected to normal vision, and none of the participants had a presence or history of psychiatric and/or neurologic comorbidities based on a clinical interview conducted by a clinical psychologist (refer to lines nineteen to twenty-eight of Table 8 for details, and for more in-depth details of this study about the design of the experiment, stimulation protocol, and data analysis methods, refer to Nejati et al. (2024)).
$$\blacksquare $$
Recognizing the importance of examining the learning and decision-making patterns of individuals with depression and anxiety, Nejati and Alavi (2024) conducted a study using the IGT. In their study, they considered two clinical groups of depression and anxiety along with a group of healthy individuals. In lines 29 and 30 of Table 8, we have grouped the combined data of anxious and depressed individuals in one line and the data of healthy individuals in another line.
$$\blacksquare $$
In another clinical study, Nejati et al. (2022) assessed the role of vmPFC and dlPFC in the outcome and process of decision-making in individuals with and without depression through IGT performance during brain stimulation. Specifically, transcranial direct current stimulation (tDCS) was conducted to alter the excitability of the respective target regions. Twenty adults with MDD (mean age 20.35± 6.83, all female) and 18 healthy adults (mean age 28.28±10, 8 female) received tDCS in three separate sessions at 72-h intervals during task performance: anodal tDCS over the left dlPFC coupled with cathodal tDCS over the right vmPFC, the reversed order of polarities, and sham stimulation. It is noteworthy that in this study, participants with MDD were diagnosed by a psychiatrist based on the Diagnostic and Statistical Manual of Mental Disorders 5th ed., as is customary. Also, all participants had severe depression based on the Beck Depression Inventory (BDI), and none of the participants had a history of head trauma, seizure, or other neurological and psychiatric disorders. Another important point included in this study is that the participants were unaware of the stimulation protocols and the aims of the study. Nejati et al. (2022) provide more detail about the subjects of this study, such as that all subjects were right-handed and had normal or corrected to normal vision. Moreover, six participants were under medication during the study (Sertraline in 2, Fluoxetine in 2, Citalopram in 1, and Bupropion in 1 participant). Furthermore, the participants did not take their medication at least 24±4 h before the stimulation session. Numbers thirty-one to thirty-six of Table 8 show the demographic parameters of the participants.
$$\blacksquare $$
The final clinical study considered for the data pool of this article is the work of Nejati et al. (2024), which investigated experience-based decision-making mechanisms in individuals with and without depression, this time during tACS. They hypothesized that the imbalanced activity of the dlPFC and the vmPFC in depression patients results in abnormal emotional information processing and that modulating it via tACS reduces biased emotional processing. Seventeen healthy adults and seventeen adults with depression received tACS in 5 separate sessions (see numbers 37 to 46 of Table 8 for details). In two stimulation conditions, two channels were used for stimulation with a relative $0^o$ “synchronized” condition or $180^o$ “desynchronized” condition phase difference. The stimulation conditions were: (a) two channels with synchronized/in-phase stimulation: a-1: F3-right shoulder and a-2: FP2-left shoulder, (b) two desynchronized/ anti-phase stimulation: b-1: F3-right shoulder and b-2: FP2-left shoulder, (c) one-channel stimulation with electrode placement over the F3 and right shoulder, (d) one-channel stimulation with electrode placement over the FP2 and left shoulder, and (e) sham condition in which a random combination of the above-mentioned electrode positions was applied in different participants, and the electrical current was ramped up for 15 s to generate the same sensation as the active conditions, and then turned off without participants’ awareness. Five minutes after the beginning of stimulation, participants performed the IGT, which lasted for about 10 min.

Based on these details mentioned above, our data pool in Table 8 lists the datasets of many laboratories used in this research.

#### Fitting Procedures

In our study, we adopted Variational Inference (VI) (Friston et al., 2007; Daunizeau et al., 2009, 2014) using CmdStan (version 2.30.0) as a strategy for data fitting. CmdStan implements Automatic Differentiation Variational Inference (ADVI) proposed by Kucukelbir et al. (2017). Unlike the Markov chain Monte Carlo (MCMC) method, which approximates the target distribution through direct sampling, VI- and therefore AVDI - simplifies the problem by utilizing Kullback–Leibler (KL) divergence (Gunapati et al., 2022). Specifically, VI minimizes the KL divergence from the variational distribution (commonly and, in our study, normal distribution) to the target posterior distribution. However, rather than minimizing the KL divergence, the evidence lower bound (ELBO) is maximized to estimate the variational posterior (Gunapati et al., 2022). AVDI also integrates the Monte Carlo method in the approximation of the ELBO. Interested readers can find the technical details of the method in the appendix.

We used the Stan programming language to develop models for ORL and VSE and implemented the VI method to approximate the posterior distribution. It is important to highlight that while Haines et al. (2018) utilized the Hamiltonian Monte Carlo No-U-Turn sampler (HMC-NUTS), a variant of MCMC, for their fitting process, we found that the chains of the HMC-NUTS sampler rarely converged for VSE model. This could be due to the local minimum problems often encountered in Bayesian inference, particularly when dealing with large datasets and equation solving Blei et al. (2017). We noticed that VSE model frequently faces this issue, leading to a failure in the convergence of chains in Bayesian inference algorithms such as HMC-NUTS.

This might explain why Ligneul (2019) chose VI using the Variational Bayesian Analysis (VBA) Matlab toolbox (Daunizeau et al., 2014) for their fitting procedure. We followed a similar strategy to address this challenge, writing both ORL and VSE models in Stan language and applying CmdStan Variational approximation to both. It is important to note that we also tested the HMC-NUTS approach for the ORL model and confirmed that the VI approach yielded superior results, thus justifying our choice of VI for both models.

We assumed that individual-level parameters were drawn from group-level distributions across all datasets in our pool. Furthermore, we assumed these group-level distributions to be normally distributed. For the ORL model, we adhered to the same means and standard deviations as in the original paper (Haines et al., 2018). However, for VSE, we set the standard deviations to 3.0, which led to improved scores and reduced divergence. Therefore, according to Haines et al. (2018), the approach for bounded parameters of ORL remains the same; for instance, for $A_{rew}$, the distribution is formed according to the following relations:

$$\begin{aligned} \mu _{A_{rew}}\sim &  Normal(0,1), \nonumber \\ \sigma _{A_{rew}}\sim &  Normal(0,.2), \nonumber \\ A_{rew}^{'}\sim &  Normal(0,1), \nonumber \\ A_{rew}= &  Probit(\mu _{A_{rew}}+\sigma _{A_{rew}} A_{rew}^{'}), \nonumber \\ K= &  Probit(\mu _{k}+\sigma _{k} A_{rew}^{'}). \nonumber \end{aligned}$$

In line with the methodology proposed by Haines et al. (2018), bounded parameters are sampled in an unconstrained space and subsequently transformed to a constrained space via a probit transformation, which is the inverse cumulative distribution function. In this context, $\mu$ represents the mean, and $\sigma$ denotes the variance of the group-level distribution. Furthermore, $A_{rew}^{'}$ is a vector that encompasses individual-level parameters in the unconstrained space, and following the probit transformation, $A_{rew}$ comprises individual-level parameters in the constrained space. Haines et al. employed a half-Cauchy (0,1) distribution for unbounded parameters for hyperstandard deviation. We adopted a similar strategy for the bounded parameters of VSE, albeit with a minor modification; we set $\mu =0$ and $\sigma =3.0$ and opted for a normal distribution instead of a half-Cauchy for unbounded parameters. Lastly, we selected the mean field algorithm and set the maximum number of iterations for ADVI to 10000 for 1000 out of the posterior samples.

The log-likelihood of each subject’s actual choice, conditioned on the parameter estimations and choices from previous trials, enables the computation of the log pointwise posterior predictive density (LPD) (Gelman et al., 2014) for a given dataset. From the log-likelihood and LPD, one can derive the AIC, BIC, and WAIC (Gelman et al., 2014), all of which are extensions of AIC. Further details of these measures can be found in the appendix for those interested.

Figure 4 illustrates the graphical Bayesian model for the hierarchical analysis of VSE and ORL, depicted in subplots *a* and *b*, respectively (see the Appendix).

**Fig. 1:**
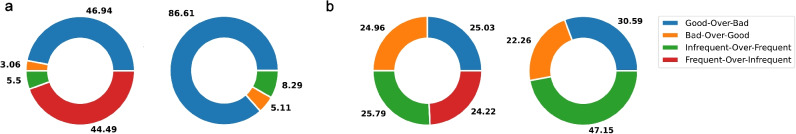
Parameter space proportions for ORL and VSE models. **a** The pair of plots demonstrates parameter space proportions of ORL according to the broad definition on the left and the restricted definition on the right. ORL exhibits an unbalanced parameter space, heavily skewed toward Good-over-Bad and Frequent-Over-Infrequent choice patterns. It can even be said that the form on the right, the limited definition, highlights this fact even more. **b** The plot on the left depicts the parameter space proportions of the VSE according to the broad definition, whereas the plot on the right concerns the restricted definition. VSE demonstrates a balanced parameter space, regardless of the restricted/broad definition used. While nearly a quarter of its parameter space generates each choice pattern according to the broad definition, Frequent-Over-Infrequent and Good-Over-Bad choice patterns dominate ORL’s major parameter space

## Results

### Simulation Perspective and Parameter Spaces of the Models

In evaluating the performance of the cognitive models, it is crucial to consider expected outcomes based on prior empirical data and existing literature. Specifically, models that can explain a diverse range of behaviors—such as favoring advantageous decks over disadvantageous ones, as well as varying preferences for loss frequency—are considered more robust and generalizable.

Empirical findings suggest that a desirable distribution of choice patterns should reflect the natural variability observed in human decision-making. For instance, healthy individuals often exhibit a “Good-Over-Bad” pattern, where advantageous decks (C and D) are chosen more frequently than disadvantageous decks (A and B). However, certain clinical populations may demonstrate different strategies, such as a “Bad-Over-Good” pattern, where disadvantageous decks are favored (Steingroever et al., 2013a). A model that can accurately reflect these patterns across its parameter space is better suited for generalizing across different datasets.

**Fig. 2:**
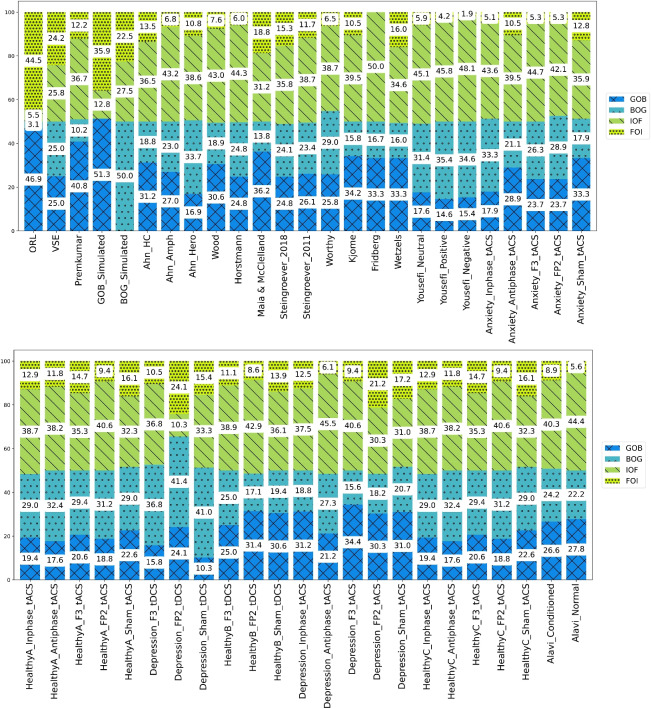
The distribution of choice patterns within our data pool, as per the broad definition. The broad definition encompasses several categories: GOB, BOG, IOF, FOI, and a category termed “Remaining” for all other potential choice patterns. Given the negligible proportion of “Remaining” choice patterns in our data pool, this category was excluded from consideration. In the upper section of the plot, the first two bars on the left represent the parameters of ORL and VSE, while the subsequent bars display the proportions of each dataset within our data pool

By establishing these benchmarks, we can assess whether the models under consideration—specifically the ORL and VSE models—achieve the expected distribution of choice patterns. As a general rule of thumb, if a model fails to generate a specific choice pattern or only does so with limited parameter sets, it is unlikely to capture that choice pattern during the fitting procedure. Conversely, datasets from real participants, whether from clinical or healthy control groups, inherently incorporate certain choice patterns. Therefore, the model fitting success is evaluated based on the correspondence between the model’s potential and the choice patterns present in the dataset. In our analysis, we used these benchmarks to evaluate the proportion of the parameter space that each model dedicates to generating the key choice patterns observed in empirical studies.

Considering that by evaluating the parameter space of the ORL and VSE models, it becomes possible to judge and predict the possibilities of related use cases, we assessed the parameter space of ORL and VSE through simulation for 777,600,000 parameter sets (derived from 60 samples across five different parameters, yielding $60^{5}$ parameter sets). Indeed, we ran the models per parameter set through simulation, with the outcome of 100 trials forming a complete IGT run for a participant. Just as a real subject behavior in the IGT yields some choice patterns, a simulated participant also displays at least one detectable choice pattern. After simulating all parameter sets, we obtained the proportions of choice patterns generated by the models Fig. 1 illustrates the parameter space of both ORL and VSE models, using both restricted and broad definitions (refer to the method section for these definitions).

Paying attention to this figure, it is evident that the parameter space of the ORL model is significantly unbalanced, as a substantial 47% of the generated patterns fall under the Good-Over-Bad category according to a broad definition. In stark contrast, a mere 3% are classified as Bad-Over-Good, and only 5% as Infrequent-Over-Frequent, while 45% are categorized as Frequent-Over-Infrequent. This suggests that the ORL model predominantly generates Good-Over-Bad and Frequent-Over-Infrequent choice patterns across most of its parameter sets. However, it only generates a small fraction of the other choice patterns, namely Bad-Over-Good and Infrequent-Over-Frequent, which are deemed experimentally necessary based on observations from clinical and control groups.

The VSE model exhibits different behavior, generating relatively equal proportions of each choice pattern, thereby demonstrating a more balanced parameter space. This is evident from the broad and restricted definition plots of this model in Fig. 1. Specifically, from a broad definition perspective, it is observed that approximately 25% of the generated patterns are Good-Over-Bad, nearly 25% are Bad-Over-Good, 25% are Infrequent-Over-Frequent, and the remaining 25% are Frequent-Over-Infrequent.

The unbalanced parameter space of the ORL model indicates a high propensity for capturing various Good-Over-Bad patterns, i.e., patterns of Deck selections where the number of cards selected from good Decks $(C + D)$ surpasses that from bad Decks $(A + B)$. Conversely, the Bad-Over-Good choice pattern occupies a considerably small portion of ORL’s parameter space, suggesting a low likelihood of capturing this pattern when fitting a dataset. This could be a significant omission, particularly if the dataset pertains to clinical groups such as those with addictions, where the Bad-Over-Good pattern is prevalent (Fridberg et al., 2010; Ahn et al., 2014; Verdejo-Garcia et al., 2006). Similarly, the Infrequent-Over-Frequent choice pattern is generated by only a small portion of the ORL’s parameter space.

**Table 4:** A comparative analysis of the parameter spaces for ORL, VSE, and all other RL models previously proposed for IGT

Choice patterns		Proportions of all choice patterns					
	EV	PVL	EV-PU	PVL-Delta	ORL	VSE	
Good > Bad	{C,D} > {A,B}	0.585	0.427	0.661	0.596	0.866	0.305
Bad > Good	{A,B} > {C,D}	0.153	0.005	0.005	0.006	0.051	0.222
Infrequent > Frequent	{B,D} > {A,C}	0.075	0.363	0.181	0.118	0.082	0.471

The restricted definition only includes three choice patterns experimentally observed in healthy participants. Notably, among these models, VSE exhibits the most balanced parameter space. The proportions of choice patterns for EV, PVL, EV-PU, and PVL-Delta, as derived here, align perfectly with the patterns reported in the study by Steingroever et al. (2013a, 2013b)

Returning to the latter portion of our findings, Fig. 2 elucidates the impact of each choice pattern on the pattern proportions of datasets within our data pool, as well as within the parameter spaces of both ORL and VSE. The relationship between a distinct choice pattern’s proportion within a dataset and its corresponding proportion within the parameter space of either ORL or VSE is a point of significant interest. As depicted in Fig. 2, a significant correspondence exists between the choice patterns present in each dataset and the model’s flexibility to generate the relevant choice patterns. In essence, the proportions of choice patterns, defined by both restricted and broad criteria, provide insightful details that further substantiate our results. For instance, nearly 10% of the choice patterns in the *Depression_sham_tDCS* dataset belong to the Good-Over-Bad category, while over 41% are classified as Bad-Over-Good. When comparing these patterns with those generated by the ORL and VSE models, it is evident that ORL generates very few Bad-Over-Good choices, with only 3% of its parameter space yielding such patterns. In contrast, more than 24% of VSE’s parameter space is dedicated to the Bad-Over-Good choice pattern, indicating that VSE is more likely to fit this dataset. This conclusion is further supported by the final results, as the AIC, BIC, and WAIC values are lower for VSE than for ORL for this dataset.

Another critical aspect to contemplate is the comparison of the proportions of chosen patterns in these two novel models with prior RL models such as EV, PVL, EV-PU, and PVL-Delta, as per the restricted and broad definitions. Tables 4 and 5 juxtapose the parameter space of the ORL and VSE models with those of previously suggested RL models for IGT, according to the restricted and broad definitions, respectively. The restricted definition confines itself to the three most frequently observed choice patterns, particularly among healthy control groups.

As shown, the VSE model exhibits the most balanced distribution of choice patterns across its parameter space among all RL models. This balance suggests that the VSE model is less biased towards generating specific patterns, making it more versatile in capturing the range of behaviors observed in various experimental conditions.

We interpret the VSE model’s ability to generate a wider variety of choice patterns—without favoring any particular strategy excessively—as a key advantage. This reduced bias implies that the VSE model is likely to generalize better across different datasets, including those with diverse population groups and varying experimental settings. In contrast, the ORL model, while effective in certain scenarios, shows a stronger bias towards particular patterns, which may limit its applicability in more diverse contexts.

This explicit interpretation of the VSE model’s reduced bias and its implications for generalizability underscores the importance of considering model flexibility when evaluating cognitive models. Therefore, the VSE model’s balanced approach to generating choice patterns makes it a more robust tool for understanding decision-making processes in the IGT. However, nearly 30% of its parameter space is allocated to the Good-Over-Bad pattern, while almost 22% is dedicated to the Bad-Over-Good choice pattern. This 22% is significant compared to other models, making the VSE model a suitable choice for clinical groups where learning deficits are common. Conversely, nearly 47% of the parameter space is attributed to the Infrequent-Over-Frequent pattern, which is observed regardless of whether the groups are healthy or clinical. For instance, in our data pool, the proportion of the Infrequent-Over-Frequent pattern, as illustrated in Fig. 2, was seldom less than 20%. We believe it is crucial for a model to have a reasonable likelihood of capturing this pattern; in other words, a substantial portion of a model’s parameter space should generate the Infrequent-Over-Frequent pattern.

**Table 5:** The choice patterns generated across the entire parameter space by each model, as per the broad definition

Choice patterns		Proportions of all choice patterns					
	EV	PVL	EV-PU	PVL-Delta	ORL	VSE	
Good > Bad	{C,D} > {A,B}	0.585	0.472	0.661	0.596	0.469	0.249
Bad > Good	{A,B} > {C,D}	0.153	0.005	0.005	0.006	0.030	0.247
Infrequent > Frequent	{B,D} > {A,C}	0.075	0.363	0.181	0.118	0.055	0.257
Frequent > Infrequent	{A,C} > {B,D}	0.099	0.003	0.003	0.005	0.444	0.242
Remaining		0.089	0.202	0.151	0.274	0.000	0.003

The proportions of choice patterns for EV, PVL, EV-PU, and PVL-Delta, as derived in this study, are entirely consistent with the patterns outlined in the research by Steingroever et al. (2013a, 2013b). Importantly, VSE exhibits the most balanced parameter space among these models

To satisfy our curiosity and clarify even more, we can examine the patterns that occur in our data pool, which includes healthy groups and various clinical groups (to see this data pool, you can refer to the “Experimental Datasets and Participants” section and Table 8). Figure 2 elaborates on these results with a broad definition of choice patterns. The first two bars represent the ORL and VSE models, while the subsequent bars correspond to the parameter space of distinct datasets from our data pool. For instance, the third and fourth bars correspond to the Premkumar (Premkumar et al., 2008) and GOB_Simulated datasets, respectively. These are two datasets where superior performance is expected from the ORL model, as suggested by the AIC, BIC, and WAIC scores, which we will explore in greater depth in the subsequent tables.

The Premkumar dataset is characterized by a higher prevalence of Good-Over-Bad patterns than Bad-Over-Good, and the GOB_simulated dataset completely lacks Bad-Over-Good patterns. These attributes make the ORL model particularly well-suited for these two datasets. The remaining bars depict the choice pattern proportions of datasets where either the VSE or ORL model is predicted to yield more accurate fitting results. A concise summary of the fitting results for each dataset can be found in Table 6, with a comprehensive explanation for each dataset available in the appendix.

In addition to the broad and restricted definitions, Romain Ligneul (Ligneul, 2019) proposed the concept of directed exploration choice strategy, also known as the Sequential Exploration SeqE Index. This strategy reflects a participant’s propensity to select three or four different Decks in three or four consecutive trials (Ligneul, 2019). The VSE model is specifically designed to encapsulate this strategy and relevant choice pattern, implying that the presence of directed exploration in datasets could influence model fitting accuracy. It is worth mentioning that the ORL model is also designed to capture various strategies, as previously explained. The fundamental characteristics of these models in accounting for choice strategies—independently or alongside choice patterns—represent a separate area of study beyond our current focus. To provide a clearer justification for the VSE results, particularly for Group B in Table 6, we have incorporated the directed exploration strategy.

Table 6, specifically the last three columns under the SeqE index label, represents the average count of detected sequential exploration choice patterns across all subjects for each dataset. The terms DE3 and DE4 represent the mean values of three and four different Deck selections chosen during three and four consecutive trials, respectively. These values are computed using a sliding window method. For instance, in the case of DE3, a sliding window of size three, encompassing three successive choices, is considered and moved one choice ahead. Consequently, every set of three consecutive choices is scrutinized for sequential exploration. DE4 employs a similar approach but with a window size of four. DE4F, on the other hand, signifies directed exploration involving four fixed-size consecutive choices.

In contrast, DE4F signifies directed exploration involving four fixed-size consecutive choices. Unlike the previous measures, DE4F does not use a sliding window; instead, it partitions choices into chunks of size four. For a subject with 100 trials, this results in 25 non-overlapping chunks. The DE choice pattern is then examined without overlap, highlighting a subject’s strong inclination toward sequential exploration. These characteristics of the dataset increase the likelihood of the VSE model achieving higher fitting accuracy compared to the ORL model.

**Table 6:** A comprehensive summary of the fitting results derived from the ORL and VSE models. The datasets are categorized into three groups based on the final outcomes

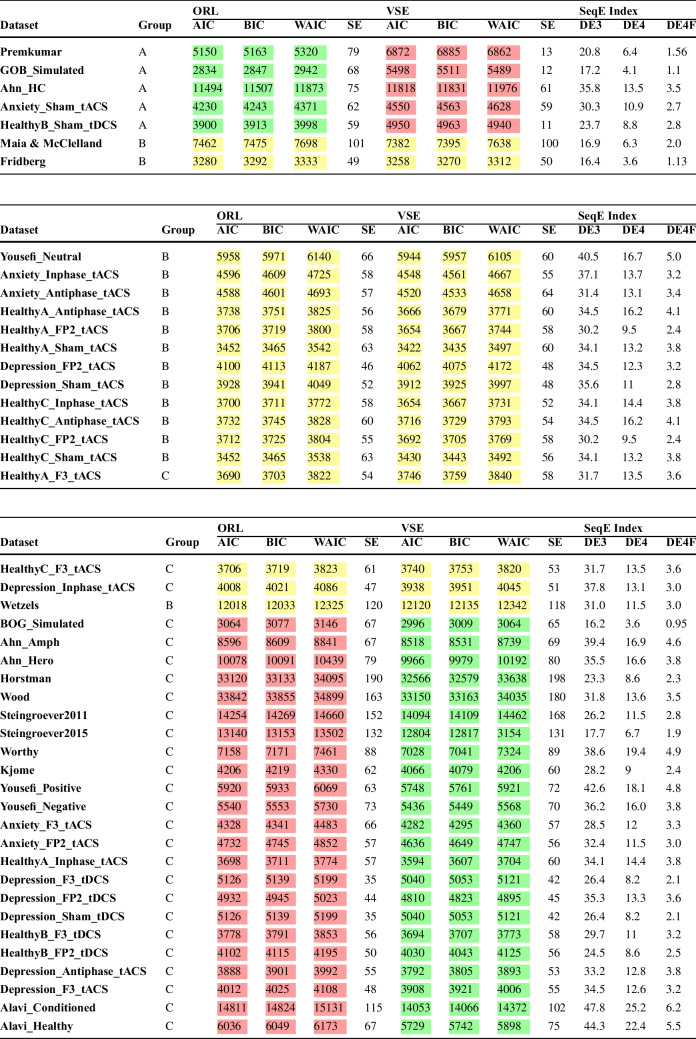	

Group A comprises datasets where the ORL model demonstrated a definitively superior result. Group B contains datasets where a decisive result could not be determined in favor of either the ORL or VSE models. Group C includes those datasets where the VSE model produced better AIC, BIC, and WAIC scores. The ORL column provides the AIC, BIC, WAIC scores, and WAIC SE (standard error) associated with the ORL model. The columns under VSE display the fitting results for each dataset using the VSE model. The SeqE index column pertains to the directed exploration choice pattern, as introduced by Romain Ligneul (Ligneul, 2019). The DE3 column represents the average number of instances where three different Deck choices were made consecutively in the relevant dataset, while DE4 indicates the average number of instances where four different Decks were chosen consecutively. Both DE3 and DE4 are calculated using a sliding window technique. Lastly, DE4F is the fixed version of DE4, implying that a subject’s choices are segmented into fixed-size chunks of 4, and the DE choice pattern is identified within these chunks. DE4F signifies a strict presence of directed exploration. To enhance readability and facilitate comparisons within the table, a color-coding system has been implemented. Light green indicates lower values, light red signifies higher values, and yellow denotes inconsistent results

**Fig. 3:**
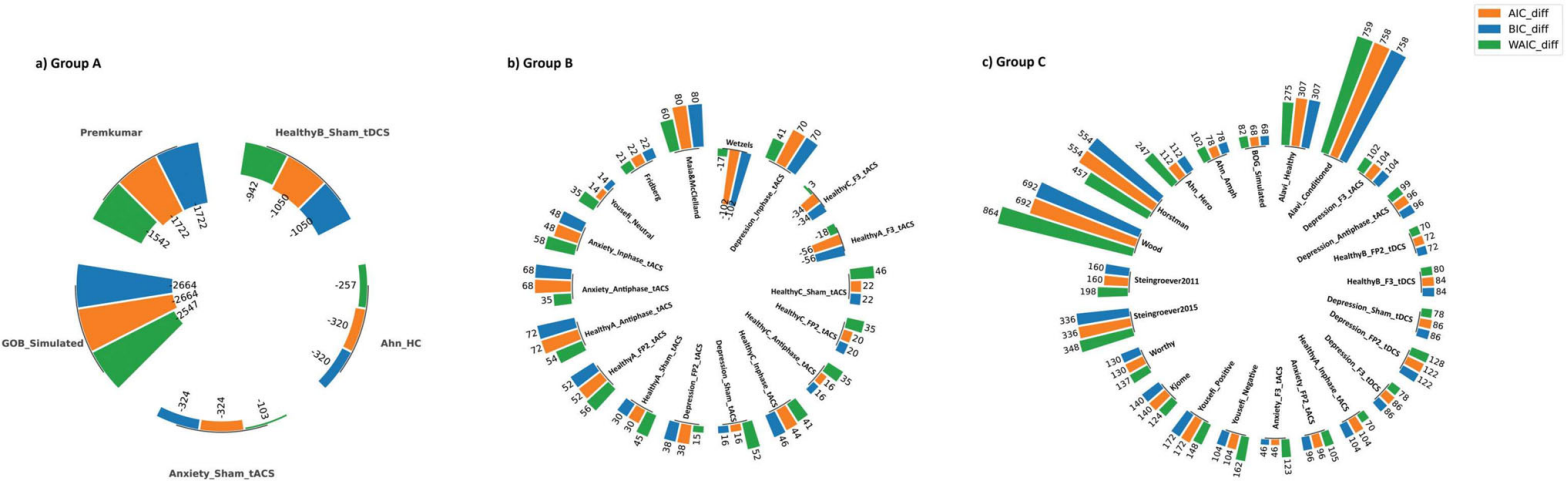
The comparison of our data pool is categorized into three groups. **a** Datasets where ORL outperformed VSE. Each bar plot displays the difference between ORL’s AIC, BIC, and WAIC values and those of VSE. A negative bar indicates that ORL’s value is smaller than VSE’s, suggesting ORL performed better. **b** Datasets for which no definitive conclusion could be reached. Here, the difference between ORL and VSE across AIC, BIC, and WAIC was smaller than the standard error, making it inconclusive. **c** Datasets where VSE outperformed ORL. In this case, a positive bar indicates that VSE’s AIC, BIC, and WAIC values are smaller, showing superior performance by VSE

### Fitting Perspective

In addition to the PSP as the most crucial model selection criterion, we utilized three additional metrics—AIC, BIC, and WAIC—to assess model performance in terms of fitting. These metrics are the primary measures many researchers depend on to compare the performance of different models (Ahn et al., 2008; Ligneul, 2019). These methods use the accuracy of models’ predictions for comparison to select the “most accurate model.” This is attainable through log predictive density or log-likelihood, as the highest log-likelihood has the highest posterior probability. Furthermore, these measures include the number of models’ parameters to compute the complexity score. A model that scores lower on these measures is deemed superior, as it indicates higher fitting accuracy while maintaining a lower level of complexity. To ensure the robustness and comprehensiveness of our results, we employed all the datasets in our data pool to fit the ORL and VSE models. This approach allows us to achieve a reliable and comprehensive result.

Figure 3 provides a comprehensive summary of the AIC, BIC, and WAIC metrics comparing the fit of the ORL and VSE models across our data pool. The results are grouped into three categories. In Fig. 3a, datasets are shown where ORL performed better, as indicated by lower AIC, BIC, and WAIC scores compared to VSE. Each dataset includes three bar plots, representing the differences between ORL and VSE for each metric. A positive value indicates that ORL had a higher score, while a negative value indicates a better fit for ORL. For example, in the *GOB_simulated* dataset, the large WAIC difference of $-$2547 strongly favors ORL, whereas in the *Anxiety_Sham_tACS* dataset, the WAIC difference is $-$103, indicating a closer fit but still sufficient for ORL to be considered the better model for this group.

On the other hand, Fig. 3b displays the datasets where the results were inconclusive. Here, the differences in AIC, BIC, and WAIC scores, while sometimes negative or positive, fall within the standard error range of the WAIC estimates for both ORL and VSE, preventing any confident determination of a superior model. Detailed information on our results is provided in Table 6.

Figure 3c contains the datasets VSE emerged as the superior model. In these datasets, all three bar plots show positive values, indicating that VSE achieved lower AIC, BIC, and WAIC scores compared to ORL. For example, the *Alavi_Conditioned* dataset shows a substantial difference in favor of VSE, while in the *Depression_F3_tDCS* dataset, the differences are smaller but still sufficient to confidently classify VSE as the better-fitting model in this group.

Table 6 provides a detailed breakdown of the AIC, BIC, and WAIC metrics for all datasets and both models, along with the SeqE index for each dataset. Consistent with Fig. 3, the final fitting results categorize the datasets into three groups: A, B, and C. The datasets in Group A indicate a preference for the ORL model based on their fitting results. Specifically, for these datasets, the AIC, BIC, and WAIC scores for the ORL model are consistently lower than those for the VSE model. To validate the WAIC results, it is evident that the discrepancy between the WAIC scores of ORL and VSE is more significant than the standard error (i.e., $|WAIC_{ORL}-WAIC_{VSE}|> max(WAIC\_SE_{ORL}, WAIC\_SE_{VSE})$).

Figure 2 illustrates that the choice patterns in Group A are predominantly characterized by Good-over-Bad and Frequent-over-Infrequent patterns, which aligns with the parameter space of the ORL model as supported by Fig. 1. This observation is further corroborated by the SeqE index (refer to the corresponding column in Table 6). A closer examination of these results reveals that the majority of datasets in Group A exhibit a lower SeqE index (associated with the DE choice pattern) compared to those in Group C. For instance, in Premkumar’s dataset, 40.8% of the pattern proportions are Good-Over-Bad, while only 10.2% are Bad-Over-Good. Given that the SeqE index (DE4F = 1.56) in this dataset is not statistically significant, it suggests that the ORL model is more likely to provide a better fit for the data.

However, the fitting results using ORL and VSE models on the datasets in Group B did not provide definitive results to ascertain the most suitable model (refer to the middle of Table 6). For instance, the relevant scores of *Wetzels, HealthyA_F3_tACS*, and *HealthyC_F3_tACS* suggest ORL’s superiority. However, the WAIC validation check rejects this conclusion. The difference between WAIC scores for the mentioned datasets falls within the standard error range; in other words, the relevant value is less than the standard error. Consequently, it is not possible to confidently assert the superiority of a model. This situation applies to several other datasets as well. Furthermore, there is no significant difference in their choice pattern proportions favoring ORL or VSE. For example, *Wetzels* choice proportions consist of 33.3% Good-Over-bad, and 16% belongs to the Bad-over-Good choice pattern. However, it appears to favor the ORL model, which it indeed does. However, because 34.6% of its choice pattern proportions belong to the Infrequent-Over-Frequent pattern, the choice pattern that comprises only 5.47% of ORL’s parameter space acts as a hindrance for ORL to confidently assert its supremacy over VSE for this dataset. Due to the diversity of characteristics of datasets in Group B, a more detailed elaboration on each result is required. The appendix provides an in-depth explanation of every dataset’s specifications and corresponding results.

On the other hand, a detailed examination of the lower half of Table 6 reveals that Group C includes datasets for which the fitting results conclusively favor the VSE model based on all three BIC, AIC, and WAIC criteria. The *Hortsmann* dataset, one of the datasets in Group C, has equal proportions (24.8%) of Good-Over-Bad and Bad-Over-Good patterns. However, the Infrequent-Over-Frequent pattern makes up 44.3% of its proportion, while ORL can scarcely capture this choice pattern (only 5.47% based on Fig. 1). Therefore, VSE is more likely to fit this dataset and capture this choice pattern (25.7% based on Fig. 1). The choice pattern proportions for all datasets are summarized in Fig. 2. A more detailed discussion of the results for each dataset is provided in the appendix.

## Discussion

Comparisons of various reinforcement learning models introduced over the past two decades to investigate the cognitive mechanisms underlying the Iowa gambling task have yielded diverse outcomes. These differences are particularly noticeable across the various datasets used for model fitting. We propose that to systematically and fairly compare the flexibility of these models, a global analysis technique is required. This approach should supersede conclusions drawn solely from local techniques, allowing us to pre-emptively understand the specific scenarios where a certain cognitive model excels, derived directly from the observed choice patterns. Here, we addressed this critical issue, utilizing PSP for a comprehensive examination of two recent RL models—the ORL and VSE models. These models were introduced to describe the underlying cognitive mechanisms in the IGT alongside older theory-driven computational models.

The latest models, ORL and VSE, each with distinct assumptions and accounting for other neurocognitive evidence, exhibit a significant degree of maturity within the RL framework. Both models demonstrate reasonable parameter recovery, simulations, and generalization test results. While the comparison approach conducted in Ligneul (2019) (Ligneul, 2019) supports the conclusion that VSE outperforms all previously proposed RL models for IGT, we contend that it is premature for a clinical experimenter to decide which model to employ for studying real-world clinical datasets. This is because a crucial aspect of the rationale for this conclusion remains unclear—the data pattern has not been considered in this conclusion. Therefore, examining this issue through both local and global lenses is necessary for a comprehensive and accurate result.

Employing the PSP method to study a model reveals its potential to generate different choice patterns. As observed in Fig. 2 in the results section, it effectively demonstrated the proportions of the choice pattern in the parameter space of ORL and VSE, as well as the dataset used in this study, using both broad and restricted definitions. The balanced parameter space of VSE enables it to capture different choice patterns of any given dataset—different sets of parameters from a model perspective—with equal probability. In contrast, ORL’s parameter space is highly unbalanced and biased. It is primarily devoted to the Good-Over-Bad and Frequent-Over-Infrequent choice patterns (with 46.94% and 44.49% in broad definition, respectively), with only a small portion generating other choice patterns (3.06% for Bad-Over-Good and 5.5% for Infrequent-Over-Frequent), which are experimentally deemed critical for a model to generate. Consequently, ORL demonstrates strong potential to capture the Good-Over-Bad and Frequent-Over-Infrequent patterns of a given dataset, while its potential to capture other choice patterns is considerably low. All in all, VSE shows higher generalizability to fit datasets with possible various choice patterns.

To be precise, the ORL model better fits datasets involving subjects with choice strategies that yield a Good-Over-Bad choice pattern (considering the restricted definition). The ORL model seems biased toward the Good-Over-Bad choice pattern, as a significant portion of its parameter space can capture various sets of parameters leading to this pattern. This flexibility makes it particularly well-suited for this type of choice behavior. However, for datasets with a high prevalence of subjects choosing Bad-Over-Good and Frequent-Over-Infrequent choice patterns, the ORL model struggles due to the restricted combinations of its parameters to generate those choice patterns. As a result, ORL has a low probability of accurately capturing the behavior of subjects producing those patterns. In contrast, the balanced parameter space of the VSE model increases the likelihood of identifying a parameter combination that effectively reproduces these patterns. The VSE model, with higher generalizability, provides equal opportunity to fit various choice patterns.

Findings suggest that different choice patterns, resulting from different strategies, may be more suited to specific components of a model than others (Steingroever et al., 2013a), and the performance of a model is dependent on the dataset (Steingroever et al., 2013a, b)Also, models’ assumptions in capturing different choice strategies play a crucial role in fitting potential. Our results showed that, even before trying to fit a model on a dataset, one can distinguish which model to choose for fitting simply by knowing the choice patterns of that dataset. However, one should not neglect the importance of models’ assumptions.

Additionally, our tests indicate that the SeqE index, a weighted indicator of the presence of a directed exploration choice pattern, slightly influences the final results. However, since the purpose of VSE is to capture this choice pattern, the SeqE index will determine VSE’s relative potential.

We present a summary of the relevant fitting results of datasets using the ORL and VSE models in Table 6. As seen there, the AIC, BIC, and WAIC measures were provided as comparative metrics for these two models, aiming to determine the most accurate fit for each dataset. The results suggest that the alignment between the available choice patterns in a dataset and the proportions of these patterns in a model’s parameter space influences which model provides a superior fit.

For datasets with a high prevalence of subjects choosing Bad-Over-Good and Frequent-Over-Infrequent choice patterns, the ORL model struggles to account for the corresponding parameter sets due to the restricted combinations of its parameters, resulting in a low probability of accurately capturing the behavior of subjects producing those patterns. In contrast, the balanced parameter space of the VSE model increases the likelihood of identifying a parameter combination that effectively reproduces these patterns.

We categorized our data pool into three groups based on these results: Group A, where ORL is the superior model with high confidence; Group B, where both models are almost equal, and it is uncertain which model is superior; and finally, Group C, which includes datasets where VSE provides a better fit. The correspondence between the pattern proportions of each dataset and the final fitting result using ORL and VSE for Groups A, B, and C can be inferred from Fig. 2 and Table 6.

In Group A, the pattern proportions align more closely with ORL’s parameter space than with VSE’s, suggesting that ORL should provide a better fit-a hypothesis confirmed by our test results (see Table 6). Conversely, in Group C, the pattern proportions of the datasets are heavily skewed toward patterns that VSE is more adept at fitting, leading us to conclude that VSE is the optimal method for these types of datasets, a conclusion supported by our test results.

Group B consists of datasets with existing patterns that do not distinctly match either model. For instance, the Good-Over-Bad choice pattern accounts for nearly 57% of Maia and McClelland’s pattern proportion, while the Bad-Over-Good pattern accounts for only 3.5%. Despite the inclination to consider ORL as a candidate model, the 39.2% Infrequent-Over-Frequent pattern presents a significant obstacle. Furthermore, the SeqE indexes of Maia and McClelland in Table 6 are relatively low compared to other datasets. Consequently, neither model can be definitively deemed superior in fitting these datasets. A detailed explanation and justification of the results for each group can be found in the appendix.

In Tables 4 and 5, we compared the parameter space proportions of the ORL, VSE, and former models. The VSE model emerged with the most balanced parameter space. Through the PSP analysis of ORL and VSE, we discerned why VSE outperformed in fitting most datasets. While the sequential exploration in a dataset, viewed as a choice strategy (Ligneul, 2019), does influence the fitting result, our findings indicate that the model’s parameter space and its alignment with the dataset used for fitting carry significantly more importance.

Consequently, VSE could potentially serve as an appropriate model for any dataset that exhibits diverse and reasonably balanced portions of choice patterns. However, it is important to note that no single model can be deemed as the universally optimal choice for all datasets, particularly for those with a substantial proportion of Infrequent-Over-Frequent or Bad-Over-Good choice patterns. The suitability of a model is heavily contingent on the choice patterns inherent in a dataset and the parameter spaces of the proposed models.

Therefore, we posit that it is of paramount importance for clinical experimenter to first conduct a thorough analysis of a dataset to identify its choice patterns. Subsequently, the model that exhibits the highest correspondence with these patterns could be selected from the available options. We believe this approach could potentially lead to more accurate and reliable results. However, further research and validation are needed to confirm this hypothesis.

## Conclusion and Future Works

Our analysis in this paper determined that the correspondence between a dataset’s choice pattern proportions and its parameter space heavily impacts the models’ fitting ability. For the RL models of the Iowa gambling task, the range of choice patterns a model can generate determines its ability to successfully capture similar choice patterns in a dataset. Despite the anticipated importance of the restricted definition for a model to capture (Steingroever et al., 2013a), our data pool analysis reveals that the Frequent-Over-Infrequent choice pattern is also empirical and influences the final fitting accuracy. These insights can benefit a clinical experimenter seeking to select the most suitable model for their dataset. To truly comprehend an IGT dataset, a clinical user should first understand its choice patterns. More specifically, they can choose a model by comparing the proportion of choice patterns in a dataset with the parameter space of various models, enabling them to achieve the most suitable fit result. It is crucial to note that this assessment’s knowledge aids in elucidating certain aspects of model comparisons. However, there is still potential for further exploration in Group B of our data pool. Future investigations should focus on the influence of each choice pattern (broad definition and SeqE index) on the final result. The weights or impacts of pattern proportions can be examined concerning each other and per model. This research line may help clarify the differences between models, particularly for Group B-type datasets in our data pool.

## Figures and Tables

**Fig. 4:**
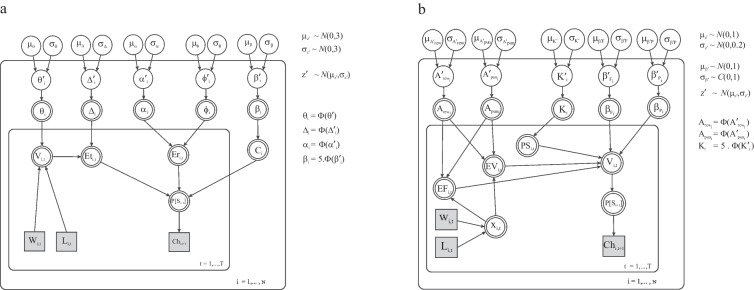
The graphical Bayesian model of ORL and VSE, designed for a hierarchical analysis. Both models are constructed following the convention established by Lee and Wagenmakers (Lee and Wagenmakers, 2014). The circular nodes symbolize the unobserved variables that are sampled within the model. The observed variables, which are available in the dataset, are represented by grey-shaded squares. The nodes depicted by double circles are deterministic variables, determined through calculation. **a** The Bayesian model for VSE, capturing the interplay of variables leading to the final choice probability. **b** The ORL model, highlighting all variables and their pertinent relationships that yield the final choice

**Fig. 5:**
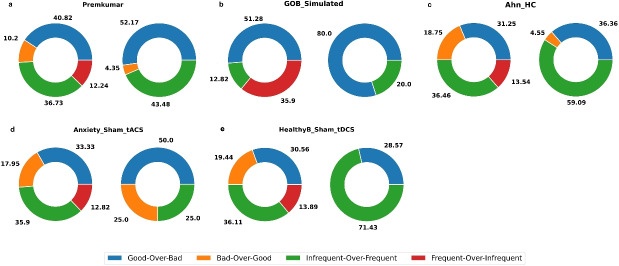
The proportions of datasets for which the ORL model yields superior fitting results. **a**, **b**, **c**, **d**, and **e** The respective proportions for the Premkumar, GOB_simulated, Ahn_HC (Healthy Control), Anxiety_Sham_tACS, and HealthyB_Sham_tDCS datasets. Each pair of plots provides a comparative analysis: the left plot delineates the choice patterns as per the broad definition, while the right plot presents the choice proportions according to the more restricted definition, encompassing 65 criteria

**Fig. 6:**
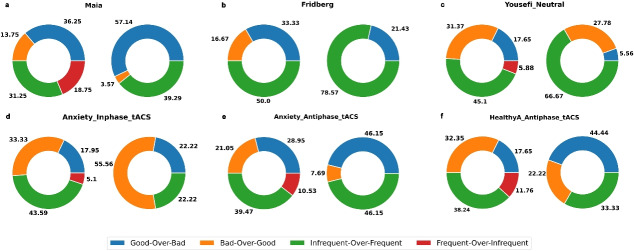
The choice pattern proportions for the first part of Group B. Preliminary fitting results in this dataset group suggest a marginal dominance of one model; however, the initial interpretation is refuted by WAIC validation checks. Consequently, a definitive conclusion remains elusive

**Fig. 7:**
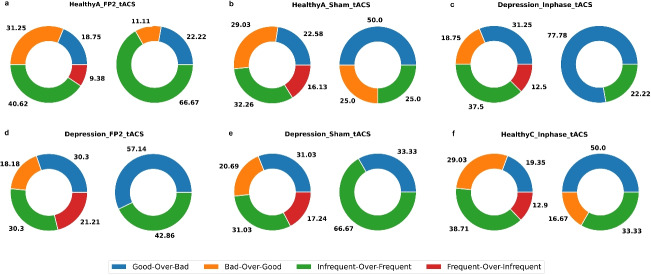
The choice pattern proportions for the second part of Group B with no conclusive fitting results

**Fig. 8:**
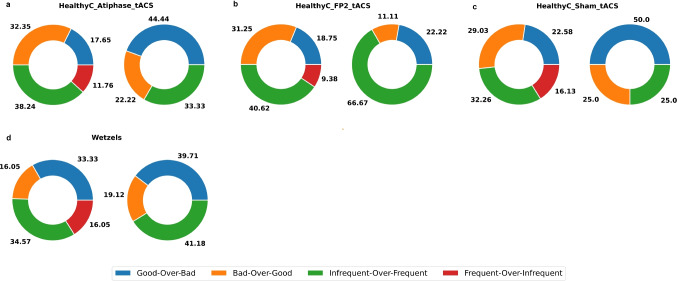
The choice pattern proportions for the third part of Group B with no conclusive fitting results

**Fig. 9:**
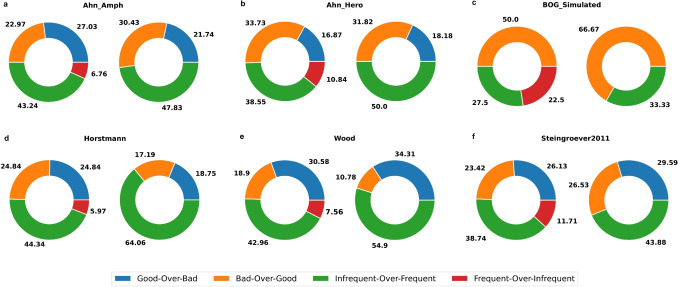
The choice pattern proportions for the first part of Group C, indicating a significantly superior fit with the VSE

**Fig. 10:**
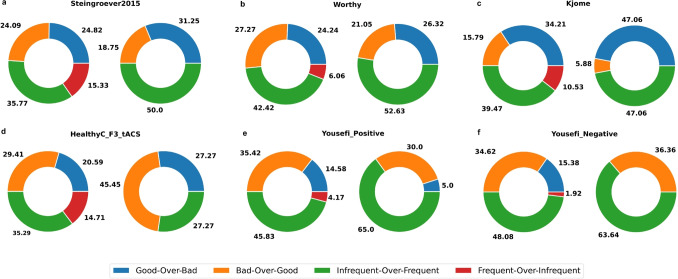
The choice pattern proportions for the second part of Group C, indicating a significantly superior fit with the VSE

**Fig. 11:**
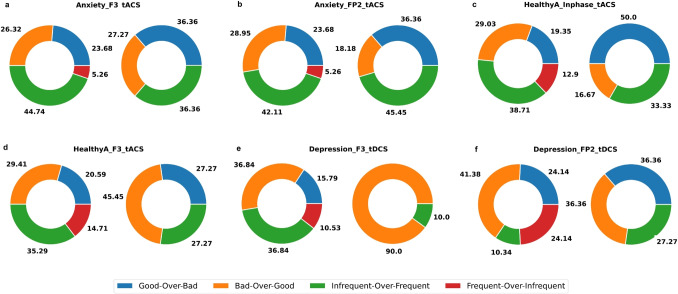
The choice pattern proportions for the third part of Group C, indicating a significantly superior fit with the VSE

**Fig. 12:**
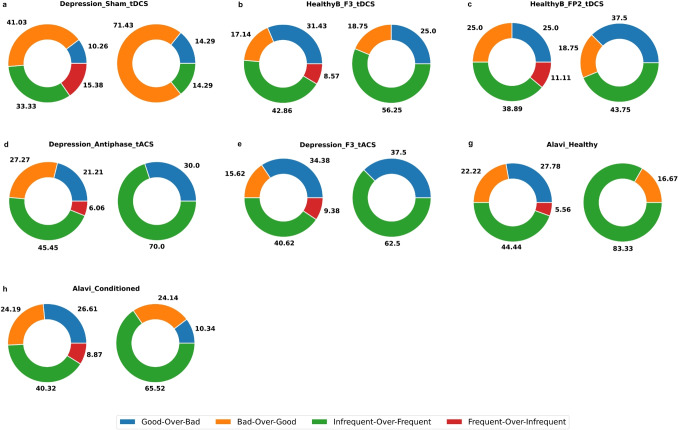
The choice pattern proportions for the fourth part of Group C, indicating a significantly superior fit with the VSE

## Data Availability

All data and simulation results generated through the PSP method are available to review in the following link on the Open Science Framework (OSF) repository: https://osf.io/zf3ku/?view_only=a1cc6a973f6e4a87baf27bb7ff259084 Additionally, the codes employed in this study are also available at the same OSF repository link for replication and further exploration. The datasets related to unpublished studies highlighted in Table 8 are available from the corresponding author upon reasonable request.

## Appendix A: Additional Model Details

Numerous RL models have been developed to identify and quantify the cognitive mechanisms underpinning the intricate behavior of the Iowa gambling task, as outlined in Table 7.

### A.1 Expectancy Valence Model

The EV model utilizes three distinct parameters: (Busemeyer and Stout, 2002; Yechiam and Busemeyer, 2005; Yechiam et al., 2005; Steingroever et al., 2013a)

- An attention weight parameter (*w*) to quantify the subjects’ attention to gains or losses ($0< w < 1$),
- An updating rate parameter (*a*) reflecting how memory is considered in learning ($0< a < 1$), with a larger value indicating rapid forgetting and a smaller value indicating a weaker recency effect or slow forgetting. In this context, “forgetting” refers to the process by which past experiences are gradually discounted in favor of more recent ones. This terminology is consistent with the original Expectancy Valence model proposed by Busemeyer and Stout (Busemeyer and Stout, 2002). It is important to note that in this context, “forgetting” does not imply the updating of unchosen options, but rather the discounting of past experiences within the decision-making process.
- A response consistency parameter (*c*) regulating sensitivity over training or the exploration-exploitation dilemma ($-5< c < 5$).

**Table 7:** A summary of previously proposed RL models, i.e. EV (Busemeyer and Stout, 2002), PVL (Ahn et al., 2008, 2013), PVL-Delta (Ahn et al., 2008; Fridberg et al., 2010; Steingroever et al., 2013b), EV-PU (Ahn et al., 2008; Steingroever et al., 2013a), and VPP (Worthy et al., 2013)

Functions	Models	Equations	Parameters	Range
Utility	EV	$u_k(t)=(1-w).W(t)+w.L(t)$	*w*: Attention weight	(0,1)
	$$\begin{array}{ll}\text {PVL} \\ \text {PVL-Delta} \\ \text {EV-PU} \\ \text {VPP} \end{array}$$	$$u_k(t)=\left\{ \begin{array}{ll}\quad \quad X(t)^{A}~~\textrm{if}~~ X(t)\geqslant 0 \\ -\lambda .|X(t)|^{A}~~\textrm{if}~~X(t)<0\end{array}\right. $$	$$\begin{array}{ll}A: \text {Shape}\\ {\lambda }: \text {Loss aversion}\end{array}$$	$$\begin{array}{ll}(0,1) \\ (0,5) \end{array}$$
Learning rule	$$\begin{array}{ll}\text {EV} \\ \text {EV-PU} \\ \text {PVL-Delta} \\ \text {VPP} \end{array}$$	$E_{v_{k}}(t)=E_{v_{k}}(t-1)+a.(u_k(t)-E_{v_{k}}(t-1))$	*a*: Updating rate	[0, 1]
	$$\begin{array}{ll}\text {PVL} \\ ~ \end{array}$$	$E_{v_{k}}(t)=a.E_{v_{k}}(t-1)+\delta _{k}(t).u_{k}(t)$	*a*: Recency	(0, 1)
Perseveration	VPP	$$\begin{array}{ll} P_{k}(t)= \Bigg \{\begin{array}{ll} d.P_{k}(t-1)+\delta _{k}(t).\epsilon _{pos}\quad \textrm{if}~~ X(t)\geqslant 0 \\ d.P_{k}(t-1)+\delta _{k}(t).\epsilon _{neg}\quad \textrm{if}~~ X(t)<0\end{array} \\ \qquad E_{v_{k}}(t)=w_{E_{v}}.E_{v_{k}}(t)+(1-w_{E_{v}}).P_{k}(t)\end{array}$$	$$\begin{array}{ll}d: Decay\\ \epsilon _{pos} \\ \epsilon _{neg} \\ w_{E_{v}}: \text {Expectancy weight}\end{array}$$	$$\begin{array}{ll}{[}0,1{]} \\ (-1,1) \\ (-1,1) \\ {[}0,1{]} \end{array}$$
Sensitivity	$$\begin{array}{ll}\text {EV} \\ \text {EV-PU} \end{array}$$	$\theta (t)=(t/10)^c$	*c* : Consistency	${[}-5,5{]}$
	$$\begin{array}{ll}\text {PVL} \\ \text {PVL-Delta} \\ \text {VPP} \end{array}$$	$\theta (t)=3^c-1$	*c* : Consistency	[0, 5]
Choice rule	All	$P{[}S_k(t+1){]}=\frac{e^{\theta (t)E_{v_{k}}(t)}}{\sum _{j=1}^{4}e^{\theta (t)E_{v_{j}}(t)}}$		

Note that $X(t) = W(t) - |L(t)|$, is the net outcome, where *W*(*t*) represents the money won and *L*(*t*) represents the money lost at time *t*. The variable *k* denotes the deck, which can take on values of 1, 2, 3, or 4 in the original version of IGT. The variable $\delta _{k}$ is a dummy variable that equals 1 if deck *k* is chosen; otherwise, 0. In the column *Range*, open intervals are denoted by parentheses, indicating that the endpoints are not included. In contrast, closed intervals are denoted by brackets, indicating that the endpoints are included

### A.2 Prospect Valance Learning Model

This model consists of four parameters:

- A shape parameter $A (0< A < 1$) determines the shape of the utility function. As *A* approaches zero, the utility function takes the form of a step function, whereas as *A* approaches 1, the utility gets close to the objective outcome amount.
- The loss aversion parameter, $\lambda (0< \lambda < 5)$. A subject with a greater loss aversion than 1 is more sensitive to losses.
- The recency parameter, $a (0< a < 1)$, quantifies the influence of Deck expectations on each trial.
- The consistency parameter, $c (0< c < 5)$, affects decision-making behavior. When $c=0$, random behavior occurs (exploration), while $c=5$ results in deterministic decision-making (exploitation)(Ahn et al., 2008; Steingroever et al., 2013a).

### A.3 Hybrid Models: VPP Model

The *Value-Plus-Perseveration* model, developed by Worthy and colleagues (Worthy et al., 2013), addresses a critical flaw in the decay rule of previous RL models. The confusion between the tendency to persist with a choice and the tendency to select the option with the highest expected value is resolved with separate terms for perseveration and expected value (Worthy et al., 2013) while maintaining other functions in common with PVL. This model is defined by eight parameters, including loss aversion, shape, and consistency parameters similar to PVL. And a decay parameter specific to the perseverance term, parameters $-1\le \epsilon _{pos}\le 1$ and $-1\le \epsilon _{neg}\le 1$ that represents the tendency to stay or switch on each trial (with positive values indicating a preference to continue selecting the same option, and negative values indicating a likelihood to switch), and the expectancy weight $0\le w_{E_{v}}\le 1$ determines the influence of expected value on decision-making, with values above 0.5 indicating a dominant expected value, and values below 0.5 indicating a stronger preservative influence.

## Appendix B: List of Datasets from Many Labs Collaborations Used in this Research

**Table 8:** List of datasets from many labs collaborations used in this research

No.	Dataset	N	Population	Study	Demographics
1	Kjome	19	Healthy	Kjome et al. (2010)	Mean (±SD) age: 33.9±11.2 years, 6 female
2	Fridberg	15	Healthy	Fridberg et al. (2010)	Mean (±SD) age: 29.6±7.6 years, 0 female
3	Wetzels	41	Healthy	Wetzels et al. (2010)	Students
4	Premkumar	25	Healthy	Premkumar et al. (2008)	Mean (±SD) age: 35.4±11.9 years, 9 female
5	Wood	153	Healthy	Wood et al. (2005)	Mean (±SD) age: 45.25±27.21 years
6	Worthy	35	Healthy	Wood et al. (2005)	Undergraduate students, 22 female
7	Maia and McClelland	40	Healthy	Maia and McClelland (2004)	Undergraduate students
8	Horstmann	162	Healthy	Steingroever et al. (2013)	Mean (±SD) age: 25.6±4.9 years, 82 female
9	Steingroever	70	Healthy	Steingroever et al. (2018)	Mean (±SD) age: 24.9±5.8 years, 49 female
10	Steingroever	57	Healthy	Steingroever et al. (2013)	Mean (±SD) age: 19.9±2.7 years, 42 female
11	Ahn_HC	48	Healthy	Ahn et al. (2014)	Mean (±SD) age: 24.7±4.9 years, 10 female
12	Ahn_Amph	38	Amphetamine	Ahn et al. (2014)	Mean (±SD) age: 22.7±3.7 years, 9 female
13	Ahn_Hero	43	Heroin	Ahn et al. (2014)	Mean (±SD) age: 29.7±5.0 years, 8 female
14	GOB_Simulated	20	−	−	−
15	BOG_Simulated	20	−	−	−
16	Yousefi_Negative	26	Negative mood induction	Yousefi and Rad (2024)	Mean (±SD) age: 22±3.83 years, 16 female
17	Yousefi_Positive	26	Positive mood induction	Yousefi and Rad (2024)	Mean (±SD) age: 22.27±2.86 years, 10 female
18	Yousefi_Neutral	26	Neutral mood induction	Yousefi and Rad (2024)	Mean (±SD) age: 23±2.9 years, 13 female
19	Anxiety_Inphase_tACS	20	Anxiety: in-phase tACS	Nejati et al. (2024)	Mean (±SD) age: 30.75±4.82 years, 16 female
20	Anxiety_Antiphase_tACS	20	Anxiety: anti-phase tACS	Nejati et al. (2024)	Mean (±SD) age: 30.75±4.82 years, 16 female
21	Anxiety_F3_tACS	20	Anxiety: F3 tACS	Nejati et al. (2024)	Mean (±SD) age: 30.75±4.82 years, 16 female
22	Anxiety_FP2_tACS	20	Anxiety: FP2 tACS	Nejati et al. (2024)	Mean (±SD) age: 30.75±4.82 years, 16 female
23	Anxiety_Sham_tACS	20	Anxiety: Sham tACS	Nejati et al. (2024)	Mean (±SD) age: 30.75±4.82 years, 16 female
24	HealthyA_Inphase_tACS	17	Healthy: in-phase tACS	Nejati et al. (2024)	Mean (±SD) age: 30±6.29 years, 10 female
25	HealthyA_Antiphase_tACS	17	Healthy: anti-phase tACS	Nejati et al. (2024)	Mean (±SD) age: 30±6.29 years, 10 female
26	HealthyA_F3_tACS	17	Healthy: F3 tACS	Nejati et al. (2024)	Mean (±SD) age: 30±6.29 years, 10 female
27	HealthyA_FP2_tACS	17	Healthy: FP2 tACS	Nejati et al. (2024)	Mean (±SD) age: 30±6.29 years, 10 female
28	HealthyA_Sham_tACS	17	Healthy: Sham tACS	Nejati et al. (2024)	Mean (±SD) age: 30±6.29 years, 10 female
29	Alavi_Conditioned	67	Conditioned	Nejati and Alavi (2024)	Mean (±SD) age: 15±2 years, 30 female
30	Alavi_Healthy	28	Healthy	Nejati and Alavi (2024)	Mean (±SD) age: 15±2 years, 13 female
31	Depression_F3_tDCS	20	Depression: F3 tDCS	Nejati et al. (2022)	Mean (±SD) age: 30.35±6.83 years, all female
32	Depression_FP2_tDCS	20	Depression: FP2 tDCS	Nejati et al. (2022)	Mean (±SD) age: 30.35±6.83 years, all female
33	Depression_Sham_tDCS	20	Depression: Sham tDCS	Nejati et al. (2022)	Mean (±SD) age: 30.35±6.83 years, all female
34	HealthyB_F3_tDCS	18	Healthy: F3 tDCS	Nejati et al. (2022)	Mean (±SD) age: 28.28±10 years, 8 female
35	HealthyB_FP2_tDCS	18	Healthy: FP2 tDCS	Nejati et al. (2022)	Mean (±SD) age: 28.28±10 years, 8 female
36	HealthyB_Sham_tDCS	18	Healthy: Sham tDCS	Nejati et al. (2022)	Mean (±SD) age: 28.28±10 years, 8 female
37	Depression_Inphase_tACS	17	Depression: in-phase tACS	Nejati et al. (2024)	Mean (±SD) age: 30.8±7.4 years, 15 female
38	Depression_Antiphase_tACS	17	Depression: anti-phase tACS	Nejati et al. (2024)	Mean (±SD) age: 30.8±7.4 years, 15 female
39	Depression_F3_tACS	17	Depression: F3 tACS	Nejati et al. (2024)	Mean (±SD) age: 30.8±7.4 years, 15 female
40	Depression_FP2_tACS	17	Depression: FP2 tACS	Nejati et al. (2024)	Mean (±SD) age: 30.8±7.4 years, 15 female
41	Depression_Sham_tACS	17	Depression: Sham tACS	Nejati et al. (2024)	Mean (±SD) age: 30.8±7.4 years, 15 female
42	HealthyC_Inphase_tACS	17	Healthy: in-phase tACS	Nejati et al. (2024)	Mean (±SD) age: 30±4.33 years, 10 female
43	HealthyC_Antiphase_tACS	17	Healthy: anti-phase tACS	Nejati et al. (2024)	Mean (±SD) age: 30±4.33 years, 10 female
44	HealthyC_F3_tACS	17	Healthy: F3 tACS	Nejati et al. (2024)	Mean (±SD) age: 30±4.33 years, 10 female
45	HealthyC_FP2_tACS	17	Healthy: FP2 tACS	Nejati et al. (2024)	Mean (±SD) age: 30±4.33 years, 10 female
46	HealthyC_Sham_tACS	17	Healthy: Sham tACS	Nejati et al. (2024)	Mean (±SD) age: 30±4.33 years, 10 female

## Appendix C: Variational Inference Methodology

VI (Friston et al., 2007; Daunizeau et al., 2009, 2014) is a technique used to approximate complex posterior distributions in Bayesian models, particularly when direct sampling methods like MCMC are computationally prohibitive. VI converts the problem of posterior inference into an optimization problem by finding a simpler distribution that closely approximates the true posterior distribution.

In our study, VI was employed to estimate the posterior distributions of parameters in the RL models analyzed. The procedure was implemented using CmdStan (version 2.30.0), which leverages ADVI (Kucukelbir et al., 2017).

### C.1 Minimization of KL Divergence

The core principle of Variational Inference is the minimization of the KL (Gunapati et al., 2022) divergence between two distributions: the variational distribution $q(\theta )$ and the true posterior distribution $p(\theta \mid \text {data})$. The KL divergence is a measure of how one probability distribution diverges from a second, expected probability distribution. Mathematically, the KL divergence from $q(\theta )$ to $p(\theta \mid \text {data})$ is defined as Friston et al. (2007); Daunizeau et al. (2009, 2014):

C.1 $$\begin{aligned} \text {KL}(q(\theta ) \parallel p(\theta \mid \text {data})) = \int q(\theta ) \log \left( \frac{q(\theta )}{p(\theta \mid \text {data})}\right) d\theta , \end{aligned}$$

where $q(\theta )$ is a chosen family of distributions that approximates the true posterior distribution $p(\theta \mid \text {data})$. The objective of VI is to find the parameters of $q(\theta )$ that minimize this divergence.

Minimizing the KL divergence effectively means making the variational distribution $q(\theta )$ as close as possible to the true posterior distribution $p(\theta \mid \text {data})$. By doing so, we obtain a distribution that can serve as an efficient and tractable approximation of the true posterior, enabling us to make inferences about the model parameters without having to directly sample from the often intractable true posterior distribution.

### C.2 ELBO and Its Role

Instead of directly minimizing the KL divergence, VI maximizes the ELBO, which is equivalent to minimizing the KL divergence in practice (Friston et al., 2007; Daunizeau et al., 2009, 2014) . The ELBO is defined as (Friston et al., 2007; Daunizeau et al., 2009, 2014):

C.2 $$\begin{aligned} \text {ELBO} = \mathbb {E}_{q(\theta )}[\log p(\text {data} \mid \theta )] - \text {KL}(q(\theta ) \parallel p(\theta )), \end{aligned}$$

where $\mathbb {E}_{q(\theta )}[\log p(\text {data} \mid \theta )]$ represents the expected log-likelihood under the variational distribution, and $\text {KL}(q(\theta ) \parallel p(\theta ))$ is the KL divergence between the variational distribution and the prior distribution over $\theta$.

By maximizing the ELBO, we effectively ensure that the variational distribution $q(\theta )$ provides a good approximation of the posterior distribution $p(\theta \mid \text {data})$, while also incorporating the prior information.

### C.3 ADVI

In this paper, we used ADVI as implemented in CmdStan. ADVI is a specific form of VI that automates the variational inference process using gradient-based optimization techniques (Kucukelbir et al., 2017). ADVI assumes that the variational distribution $q(\theta )$ belongs to the family of normal distributions, parameterized by a mean vector $\mu$ and a standard deviation vector $\sigma$. The ELBO is then optimized with respect to these parameters using gradient ascent, where the gradients are computed via automatic differentiation. This approach allows ADVI to efficiently scale to large and complex models.

The use of ADVI integrates Monte Carlo sampling into the approximation of the ELBO, ensuring that the optimization process is both accurate and computationally feasible.

For our analysis, VI was applied to both the ORL and VSE models. The variational distributions $q(\theta )$ were optimized using ADVI to approximate the true posterior distributions of the model parameters.

Once the variational distributions were obtained, we used them for posterior inference, generating estimates for the parameters that describe the cognitive processes modeled in the IGT.

## Appendix D: Supplementary Model Comparison Metrics

AIC, BIC, and WAIC serve as mathematical methodologies for evaluating the fitting results of two distinct models. AIC and BIC incorporate the number of independent variables or parameters and the maximum likelihood estimate to compute model complexity. On the other hand, WAIC employs log-likelihood and LPD to generate the pointwise WAIC and the standard error. AIC is a comprehensive method grounded in the Kullback–Leibler (KL) information loss or likelihood framework (Burnham and Anderson, 2004). As per Akaike (Akaike, 1998), the AIC can be calculated using the following equation:

D.1 $$\begin{aligned} AIC=-2 \sum _{i=1}^{n}\log p(y_{i}|\hat{\theta }_{mle})+2k, \end{aligned}$$

where $\hat{\theta }_{mle}$ represents the maximum likelihood estimate and *k* denotes the number of estimated parameters. $p(y_{i}|\hat{\theta }_{mle})$ is the pointwise predictive density for the *i*-th observation given the estimated parameters $\hat{\theta }_{mle}$. *k* is the number of parameters in the model. *n* is the numebr of observation, $y_{i}$ is the *i*-th observation. Schwarz (1978) introduced the BIC, which is motivated by the Bayes factor, and can be calculated using the following equation:

D.2 $$\begin{aligned} BIC=-2\sum _{i=1}^{n}\log p(y_{i}|\hat{\theta }_{mle})+k\log (n) \end{aligned}$$

Contrary to AIC, the correction term in BIC is changed to $\log (n)$, where *n* is the sample size of the training set or the number of observations, resulting in a larger penalty per parameter for large datasets. Note that in our study, the calculation of the AIC and BIC was based on the MLE obtained through our implementation of ADVI. To ensure the accuracy of these calculations, we removed prior distributions during the likelihood estimation process. This approach allowed us to obtain point estimates for the parameters independently of any prior assumptions, ensuring that the AIC and BIC values reflect the data-driven goodness-of-fit of the models. Specifically, the mean or mode of the approximate posterior distributions obtained through Mean-Field VI was used as a point estimate for these calculations.

Another fully Bayesian approach, WAIC, introduced by Watanabe and Opper (2010), employs log point-wise posterior density and includes a correction for the number of parameters to avoid model overfitting (Gelman et al., 2014). The WAIC score for each model is calculated using the following equation:

D.3 $$\begin{aligned} WAIC=2\sum _{i=1}^{n}(\log (E_{posterior}p(y_{i}|\theta ))-E_{posterior}(\log ~p(y_{i}|\theta )). \end{aligned}$$

## Appendix E: Graphical Bayesian Model for the Hierarchical Analysis of VSE and ORL

Figure 4 illustrates the graphical Bayesian model for the hierarchical analysis of VSE and ORL, depicted in subplots *a* and *b*, respectively. Each subplot contains two plates representing subjects and trials. The outer plate encompasses the variables and sampling processes for each subject in the IGT, while the inner plate encapsulates per-trial calculations for a specific subject. As demonstrated in Fig. 4a, individual-level parameters for subject *i* are denoted as $z_{i}$, where $z_{i}$ comprises $\{\theta _{i},\Delta _{i},\alpha _{i},\phi _{i},\beta _{i}\}$ for the VSE model. Similarly, in Fig. 4b, the parameters for the ORL model are represented by $z_{i}=\{A_{rewi},A_{puni},K_{i},\beta _{Fi},\beta _{Pi}\}$. These parameters undergo a *probit* transformation to obtain $z'_{i}=\{\theta '_{i},\Delta '_{i},\alpha '_{i},\phi '_{i},\beta '_{i}\}$ for the VSE and $z'_{i}=\{A'_{rewi},A'_{puni},K'_{i},\beta '_{Fi},\beta '_{Pi}\}$ for the ORL. The group-level normal distribution with mean $\mu _{z'}$ and standard deviation $\sigma _{z'}$ is used to sample the parameters $z'_{i}$. Notably, in the ORL model, the standard deviation for individual-level parameters $\beta _{F}$ and $\beta _{P}$ is sampled from a half-Cauchy distribution, while the standard deviation of other parameters follows a normal distribution. Both models incorporate the cumulative standard normal distribution function, denoted as $\Phi$, which serves as the inverse of the probit transformation. The sampled parameters are subsequently utilized in the equations described in Tables 3 and 2 for each trial (inner plate) to generate the next trial choice $Ch_{i,t+1}$. Additionally, $W_{i,t}$ and $L_{i,t}$ represent the reward and loss amounts received by subject *i* in trial *t*. In Fig. 4a, $V_{i,t}$, $Et_{i,t}$, $Er_{i,t}$ and $P[S_{t+1}]$ represent the utility, exploitation, exploration, and choice rule functions of the VSE model, respectively. On the other hand, the inner plate of the ORL model includes variables such as $X_{i,t}$ (utility function), $EF_{i,t}$ (expected frequency of each Deck), $EV_{i,t}$ (expected value of each Deck), $PS_{i,t}$ (perseverance tendency of each Deck), $V_{i,t}$ (single value signal), and $P[S_{t+1}]$ (choice rule of the ORL). All these functions are deterministic and are computed accordingly.

## Appendix F: Datasets Choice Pattern Proportions

A comprehensive analysis of 45 datasets from our data pool provides valuable insights into the fitting outcomes of ORL and VSE models. We meticulously evaluated the choice patterns in each dataset, applying both broad and restricted definitions, incorporating 65 distinct criteria. As detailed in the results section of the paper, Table 6 categorizes the findings into three groups. Group A includes results that affirm the ORL model, while Group B contains those where it is unclear which model is superior. On the other hand, Group C consists of datasets where the fitting outcomes indicate a clear advantage of VSE over ORL. Figures 5 to 12 provide a detailed visual representation of the choice pattern proportions across these groups.

Figure 5 provides a visual representation of the choice proportions made by Group A, with a particular focus on the data sets where the ORL model demonstrated superior fitting. Specifically, Fig. 5a displays the proportions of choice patterns for Premkumar, delineated by both restricted and broad definitions (displayed on the right and left, respectively). It is evident that the Good-Over-Bad pattern proportion is nearly quadruple that of the Bad-Over-Good patterns. This disparity is further highlighted when random behavior is excluded from the definition. A closer look at Fig. 1 reveals that the ORL model is more proficient in capturing the Good-Over-Bad pattern. Given that the Bad-Over-Good patterns are less prevalent in this data set, the corresponding AIC, BIC, and WAIC values of the ORL and VSE models for the Premkumar data set, as listed in Table 6, seem reasonable. Moreover, the incidence of the directed exploration (also referred to as sequential exploration) choice pattern is significantly lower compared to the other data sets in our data pool, which further validates the final results derived from the ORL model.

Figure 5b shows the proportions of choice patterns for GOB_Simulated, a data set we specifically generated to examine the sensitivity of both ORL and VSE models towards Good-Over-Bad and Bad-Over-Good patterns. As expected, the ORL model outperformed the VSE model in terms of relevant scores, as detailed in Table 6. Interestingly, this figure reveals the absence of Good-Over-Bad choice patterns, under both broad and restricted definitions. Furthermore, the AIC, BIC, and WAIC scores for the ORL model are nearly half of those for the VSE model in the context of the GOB_Simulated data set. These findings align with the corresponding results in terms of SE indices.

On the other hand, looking at the third plot in Fig. 5, it can also be seen that the choice pattern proportions of Ahn_HC closely mirror those of Premkumar. Once again, the Bad-Over-Good portion is significantly smaller compared to Good-Over-Bad, particularly under the restricted definition. Despite the SeqE index in Ahn_HC being nearly twice as large as that for Premkumar, the ORL model still produced a superior fit (refer to Table 6 for details). These findings imply that the proportions of Bad-Over-Good and Good-Over-Bad may have a greater impact on the outcome than the SE choice patterns.

In addition, it is worth noting explicitly that the choice pattern propositions for the Anxiety_Sham_tACS, depicted in subplot *d*, demonstrate a clear dominance of the Good-Over-Bad choice pattern. This dominance is even more pronounced in the restricted definition (plot on the right). The SeqE index of this dataset is also low, further supporting the final result of ORL supremacy.

Moreover, Group A includes the HealthyB_Sham_tDCS dataset, which exhibits a choice proportion strikingly similar to Anxiety_Sham_tACS as per the broad definition (left-hand plots of subplots *d* and *e*). Upon excluding random behavior from the dataset, all BOG patterns are consequently eliminated, indicating the absence of a stringent Bad-Over-Good preference for this dataset. As per the SeqE indexes in Table 6, the lower AIC, BIC, and WAIC scores of ORL are apparent.

Figure 6 illustrates a graphical representation of the first part of Group B from our data pool. This group contains datasets for which the WAIC validation test did not corroborate the preliminary conclusions drawn from the fitting process. Consequently, even though one model appeared to have a slight edge, its presumed dominance was not validated. Figure 6a depicts the distribution of choice patterns for the *Maia and McClelland* dataset, as per both the broad (left) and the restricted definition 65 criteria (right). Despite the lower AIC, BIC, and WAIC scores of the VSE model for this dataset, suggesting its superiority over the ORL model (refer to Table 6), the discrepancy in the WAIC scores of the two models significantly exceeds the WAIC SE of either model. This is justified by the alignment between the choice patterns in the *Maia and McClelland* dataset and the parameter spaces of the ORL and VSE models (see Fig. 1). It is worth noting that the dominant choice patterns in the ORL parameter space are Good-Over-Bad and Frequent-Over-Infrequent, while the *Maia and McClelland* dataset primarily exhibits Good-Over-Bad and Infrequent-Over-Frequent patterns. Furthermore, the SeqE index for this dataset is relatively low, aligning with the choice pattern that the VSE model aims to capture (see Table 6). Therefore, it can be concluded that both models demonstrate comparable potential in terms of performance.

Given the similarity of other data patterns in this group to the aforementioned items, we will forego a detailed explanation of all of them and focus on a select few. For instance, Fig. 6b represents the choice pattern proportion of the *Fridberg* dataset. Both the broad and restricted plots reveal that the Bad-Over-Good pattern is not a major choice pattern, while Infrequent-Over-Frequent constitutes nearly 80% of the patterns. Additionally, the SeqE index for this dataset is quite low (see Table 6), leading to an inconclusive fitting result for VSE. As another example in this group, Fig. 6e displays the proportions of choice patterns in the *Anxiety_Antiphase_tACS* dataset. Similarly, the pattern proportions for ORL and VSE are almost identical, indicating similar odds. Lastly, in the *HealthyA_Antiphase_tACS* dataset, VSE appears to yield a better-fitting score initially, but not by a conclusive margin over ORL. The key lies in the pattern proportions, where both models are almost equally favored.

Figure 7 provides a comprehensive summary of the choice pattern proportions for the second part of the Group B dataset. A closer examination of Fig. 7a, which depicts the pattern proportions of *HealthyA_FP2tACS*, reveals that the VSE index seems to be more effective in encapsulating the available patterns. This is particularly evident when considering the restricted definition that underscores the prevalence of Bad-Over-Good and Infrequent-Over-Frequent patterns. However, it is worth noting that the SeqE index for this dataset is relatively small.

On the other hand, Fig. 7b pertains to the choice patterns of the *HealthyA_Sham_tACS* dataset. Our observations reveal that approximately 50% of the data is allocated to Good-Over-Bad, while the remaining 50% is distributed between Infrequent-Over-Frequent and Bad-Over-Good, as per the restricted definition that underscores this correlation. Consequently, it is reasonable to assume that both models have comparable probabilities of achieving a good fit.

As the final look at Fig. 7, we observe a comparable distribution of choice pattern proportions across the *Depression_Inpahse_tACS*, *Depression_FP2_tACS* and *Depression_Sham_tACS* datasets. The broad plots demonstrate that the fitting results for these datasets were inconclusive, as the choice patterns were almost evenly split between both models. According to the restricted definition, all of these datasets feature Bad-Over-Good patterns. However, the proportion of Infrequent-Over-Infrequent patterns is notably high in all of them, contributing a negligible portion to ORL’s pattern proportion. Interestingly, Fig. 7f reveals that the *HealthyC_Inphase_tACS* dataset contains precisely equal proportions of Good-Over-Bad, Bad-Over-Good, and Infrequent-Over-Frequent, suggesting a nearly equal likelihood for both models.

The pattern proportions of three additional datasets from Group B, namely *HealthyC_Antiphase_tACS*, *HealthyC_FP2**_tACS*, and *HealthyC_Sham_tACS*, as depicted in Fig. 8 a, b, and c, can be interpreted similarly. The major patterns in the ORL parameter space, which favor the ORL model (Good-Over-Bad), are nearly equivalent to Bad-Over-Good and Infrequent-Over-Infrequent. This equivalence explains the inconclusive fitting result, as shown by the restricted definition plot, with the exception of b. However, the pattern proportions for *HealthyC_FP2_tACS* appear to be primarily Bad-Over-Good and Infrequent-Over-Infrequent. Despite this, its relatively low SeqE index renders it unsuitable for the VSE.

In comparison to the ORL model, the data sets in Group C demonstrate a superior fit for the VSE model, as evidenced by the AIC, BIC, and WAIC measures. The pattern proportions across all datasets in this group align with the dominance of the VSE model. Figure 9 reveals that the Infrequent-Over-Frequent and Bad-Over-Good patterns are prevalent, which accounts for the lower AIC, BIC, and WAIC scores of VSE. Despite the pattern proportions in Fig. 9(*b*) for the *Ahn_Hero* data set being akin to those of *HealthyC_FP2_tACS* (refer to Fig. 8b), the elevated SeqE index of *Ahn_Hero*, particularly the DE3 score, leads to the VSE model outperforming the ORL model. It is important to note that the VSE model is specifically designed to capture the sequential exploration choice pattern.

Figure 10 illustrates the second part of Group C. The pattern proportions in subplots a–f underscore the dominance of patterns that lend the VSE model a decisive edge over the ORL model. While the ORL model struggles to capture the Bad-Over-Good and Infrequent-Over-Frequent patterns, these patterns are predominant across all data sets in Group C. It is worth noting that significant disparities exist between pattern proportions in some of these data sets, such as *Yousefi_Positive*. Consequently, the VSE model yields lower AIC, BIC, and WIAC scores compared to the ORL model.

Figure 11 provides a summary of the pattern proportions for the third part of Group C. Take, for instance, Fig. 11a, which illustrates the pattern proportions for *Anxiety_F3_tACS*. The majority of the patterns are Bad-Over-Good and Infrequent-Over-Frequent, accounting for 71.06% in the broad definition and 63.63% in the restricted definition. As a result, the AIC, BIC, and WAIC scores for the VSE model are lower than those for the ORL model (please see Table 6 for a detailed explanation that follows the same rationale).

Figure 12 provides a summary of the pattern proportions for the remaining data sets in Group C. In this context, the proportions of choice patterns are significantly influenced by the Bad-Over-Good and Infrequent-Over-Frequent choices, which pose a challenge for the ORL model to capture (please refer to the ORL parameter space in Fig. 1 for more details). As depicted in Fig. 12 d and e, the plots for the restricted definition (the doughnut plots positioned to the right of each subplot) suggest that random behavior is excluded. Consequently, while the plot does not display a Bad-Over-Good pattern, a substantial part of the plot is still dedicated to the Infrequent-Over-Frequent pattern.

## Code Availability

The codes employed in this study are also available at the same OSF repository link for replication and further exploration. The datasets related to unpublished studies highlighted in Table 8 are available from the corresponding author upon reasonable request.
